# Mediator kinase inhibition reverses castration resistance of advanced prostate cancer

**DOI:** 10.1172/JCI176709

**Published:** 2024-03-28

**Authors:** Jing Li, Thomas A. Hilimire, Yueying Liu, Lili Wang, Jiaxin Liang, Balazs Gyorffy, Vitali Sikirzhytski, Hao Ji, Li Zhang, Chen Cheng, Xiaokai Ding, Kendall R. Kerr, Charles E. Dowling, Alexander A. Chumanevich, Zachary T. Mack, Gary P. Schools, Chang-uk Lim, Leigh Ellis, Xiaolin Zi, Donald C. Porter, Eugenia V. Broude, Campbell McInnes, George Wilding, Michael B. Lilly, Igor B. Roninson, Mengqian Chen

**Affiliations:** 1 Department of Drug Discovery and Biomedical Sciences, College of Pharmacy, University of South Carolina, Columbia, South Carolina, USA.; 2 Senex Biotechnology Inc., Columbia, South Carolina, USA.; 3 Division of Hematology-Oncology, Medical University of South Carolina, Charleston, South Carolina, USA.; 4 Department of Bioinformatics, Semmelweis University, Budapest, Hungary.; 5 Department of Biophysics, Medical School, University of Pecs, Pecs, Hungary.; 6 Center for Prostate Disease Research, Murtha Cancer Center Research Program, Department of Surgery, Uniformed Services University of the Health Sciences; Walter Reed National Military Medical Center; Henry M. Jackson Foundation for the Advancement of Military Medicine Inc.; Bethesda, Maryland, USA.; 7 Genitourinary Malignancies Branch, Center for Cancer Research, National Cancer Institute, National Institutes of Health, Bethesda, Maryland, USA.; 8 Departments of Urology and Pharmaceutical Sciences, University of California, Irvine, California, USA.

**Keywords:** Oncology, Therapeutics, Transcription

## Abstract

Mediator kinases CDK19 and CDK8, pleiotropic regulators of transcriptional reprogramming, are differentially regulated by androgen signaling, but both kinases are upregulated in castration-resistant prostate cancer (CRPC). Genetic or pharmacological inhibition of CDK8 and CDK19 reverses the castration-resistant phenotype and restores the sensitivity of CRPC xenografts to androgen deprivation in vivo. Prolonged CDK8/19 inhibitor treatment combined with castration not only suppressed the growth of CRPC xenografts but also induced tumor regression and cures. Transcriptomic analysis revealed that Mediator kinase inhibition amplified and modulated the effects of castration on gene expression, disrupting CRPC adaptation to androgen deprivation. Mediator kinase inactivation in tumor cells also affected stromal gene expression, indicating that Mediator kinase activity in CRPC molded the tumor microenvironment. The combination of castration and Mediator kinase inhibition downregulated the MYC pathway, and Mediator kinase inhibition suppressed a MYC-driven CRPC tumor model even without castration. CDK8/19 inhibitors showed efficacy in patient-derived xenograft models of CRPC, and a gene signature of Mediator kinase activity correlated with tumor progression and overall survival in clinical samples of metastatic CRPC. These results indicate that Mediator kinases mediated androgen-independent in vivo growth of CRPC, supporting the development of CDK8/19 inhibitors for the treatment of this presently incurable disease.

## Abstract 2

Inhibition of Mediator kinases CDK19 and CDK8 suppresses androgen-independent *in vivo* growth of castration-resistant prostate cancers via castration-induced transcriptional reprogramming in tumor and stromal cells.

## Graphical abstract

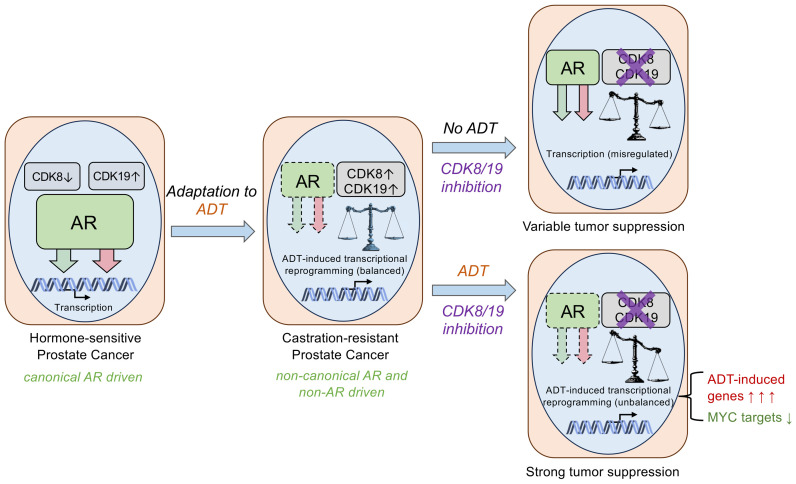

## Introduction

Androgen deprivation therapy (ADT) is the mainstay of treatment for prostate cancer (PCa), the most common cancer and the second leading cause of cancer-related mortality in men in the United States (1). However, most patients with aggressive PCa become resistant to ADT and develop castration-resistant prostate cancer (CRPC). Newer CRPC treatments targeting the androgen receptor (AR) or androgen production, such as enzalutamide and abiraterone, extend CRPC survival by only 2–8 months, and CRPC remains an incurable disease (2). Multiple mechanisms of ADT resistance have been identified, such as increased AR expression, mutations of AR ligand-binding domain, and production of androgen-independent AR variants, as well as changes in TP53, RB1, and ETS family (3). It is now understood that cancer treatment not only selects mutant therapy-resistant cells, but also induces non-genetic adaptation at the transcriptional level, leading to drug resistance (4). Transcriptional mechanisms of ADT resistance are prominent in PCa, where multiple resistance mechanisms coexist in the same tumor owing to the high heterogeneity of AR expression and of other key PCa drivers (3), and where the tumor microenvironment acts as another determinant of ADT resistance (5, 6).

CDK8 and CDK19 Mediator kinases are alternative enzymatic components of the kinase module that regulates the transcriptional Mediator complex. The Mediator kinase module includes, in addition to CDK8 or CDK19, their binding partner cyclin C (CCNC), as well as MED12/MED12L and MED13/MED13L (7). CDK8 and CDK19 paralogs have qualitatively similar effects on protein phosphorylation and transcription, but the expression of CDK8 and CDK19 is differentially regulated (8). CDK8/19 kinase activities regulate transcription both positively, by potentiating the induction of gene expression by various signals and stressors (8–11), and negatively; the latter effect involves posttranscriptional regulation of multiple proteins comprising the transcription-enhancing Mediator complex (8). Mediator kinases have been identified as broad-spectrum positive regulators of transcriptional reprogramming (8, 10, 11), but they also act as negative regulators of chemically induced reprogramming of cell fate (12). Such negative regulation provides the mechanism of the antiproliferative effect of Mediator kinase inhibition (MKI) in acute myeloid leukemia (AML) (13), where MKI led to hyperactivation of genes associated with super-enhancers (which are enriched in the Mediator complex). Remarkably, both the inhibition and hyperactivation of such genes inhibited AML cell proliferation, indicating that Mediator kinase was required for the balanced expression of the super-enhancer–associated genes (13).

Since transcriptional reprogramming is the key process in tumor cell adaptation to a heterologous tissue environment (metastasis) and to the survival of therapy, CDK8/19 inhibitors (CDK8/19i) were found to suppress the growth of metastatic tumors preferentially to primary tumors in colon cancer (14) and to prevent tumor adaptation and even overcome the acquired resistance to different classes of anticancer agents, in vitro and in vivo (15–19). In addition to their effects on tumor cells, CDK8/19i were also shown to stimulate tumor surveillance by NK cells (20, 21) and effector T cells (22). Although systemic toxicity was reported for two CDK8/19i (23), it was subsequently found to be due to off-target effects of these compounds (24). Several CDK8/19i have reached clinical trials in solid tumors and leukemias (ClinicalTrials.gov NCT03065010, NCT04021368, NCT05052255, NCT05300438) (25).

Mediator kinases show remarkable clinical correlations in PCa, the only type of cancer marked by high CDK19 expression in primary tumors (26). CDK19 in PCa correlates with Gleason grade, T stage, Ki67 index, nuclear AR expression, and ERG status (27) and can be used as a marker for the detection of advanced PCa (28). Both CDK19 and CDK8 are elevated in metastatic CRPC (mCRPC) (26, 27), and the levels of CDK19 (26) and CDK8 (29) show negative correlations with disease-free survival. CDK8/19-inhibiting small molecules have been reported to inhibit the proliferation and suppress invasive growth of some PCa cell lines (26, 30–32). However, the role of Mediator kinases in PCa in general or CRPC in particular remains unknown.

In the present study, we used genetic modifications of CDK19 and CDK8 and selective CDK8/19i to investigate the effects of MKI on androgen-independent growth of cell line–based and patient-derived xenograft (PDX) models of CRPC. We found that MKI reverses the castration-resistant phenotype of CRPC tumors in vivo, by altering the transcriptional effects of castration on both tumor and stromal genes, whereas prolonged administration of a CDK8/19i induces tumor regression and cures. We also found that the combination of castration and MKI downregulates the MYC pathway, and a MYC-driven CRPC model responds to MKI even without castration. These results support the development of CDK8/19i, a new class of nontoxic small-molecule anticancer drugs, for the treatment of the presently incurable CRPC.

## Results

### Differential effects of androgen signaling on CDK8 and CDK19 expression and upregulation of Mediator kinase module in mCRPC.

**Figure 1:**
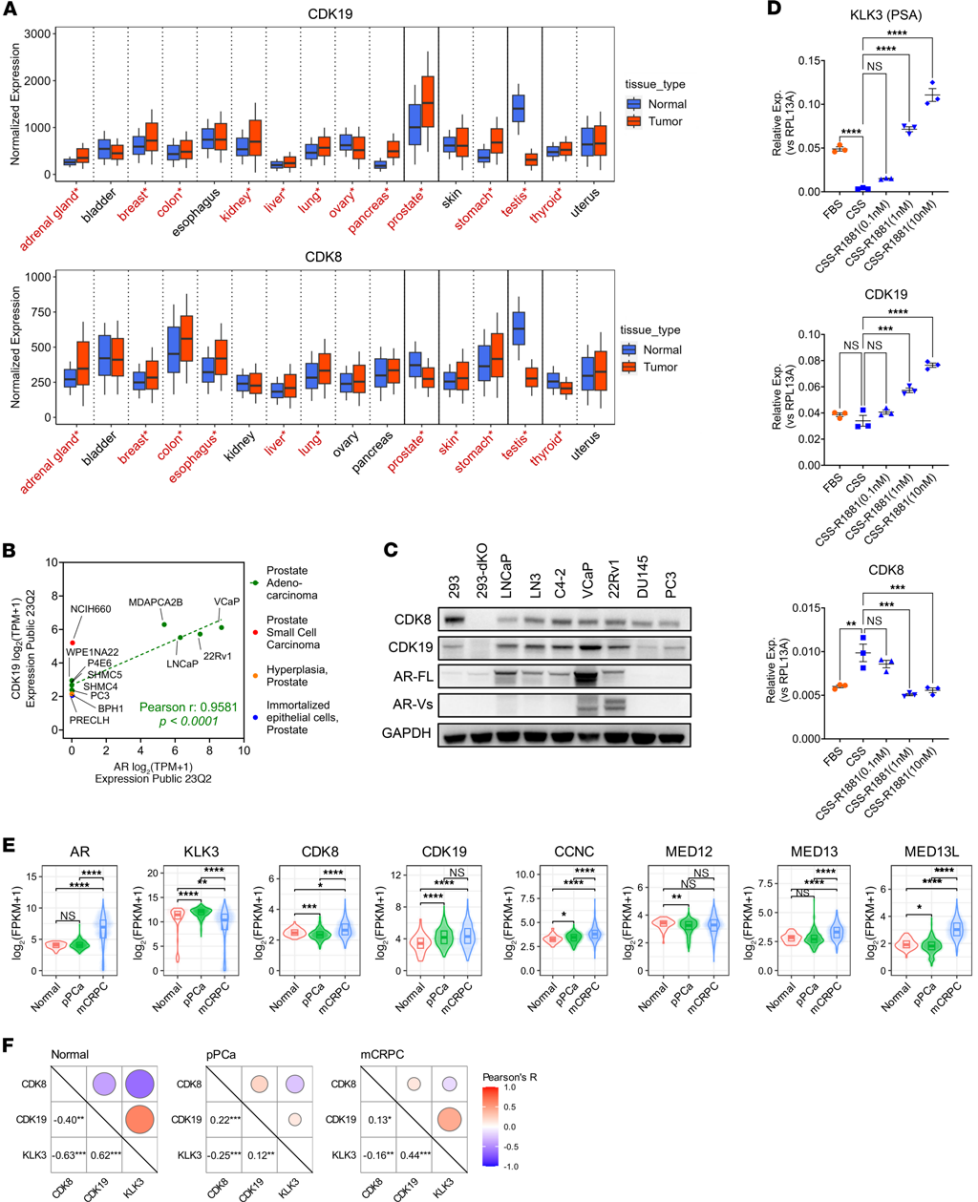
Expression of CDK19, CDK8, and their associated genes in PCa. (**A**) CDK19 and CDK8 RNA expression in normal and tumor tissues. Significant differences between normal and tumor tissues (Mann-Whitney *U* test) are marked in red and with asterisks. (**B**) RNA expression of CDK19 and AR in 12 prostate-lineage cell lines from the DepMap database. Pearson’s correlation analysis was performed for prostate adenocarcinomas (green dots). (**C**) Western blot analysis of CDK8, CDK19, AR, and GAPDH (control) in different prostate PCa cell lines and in 293 cells and their CDK8/19-double-knockout (dKO) derivative. (**D**) qPCR analysis of KLK3 (PSA), CDK19, and CDK8 RNA in LNCaP cells growing in androgen-containing (FBS) or androgen-deprived (CSS) medium, with or without 24-hour treatment with R1881 androgen at indicated concentrations. (**E**) RNA expression of AR, KLK3, and Mediator-associated CDK module subunits in normal prostate (*n* = 52), primary PCa (*n* = 502), and mCRPC (*n* = 266). (**F**) Correlation analysis between CDK8, CDK19, and KLK3 expression in normal prostate, primary PCa, and mCRPC. *P < 0.05, **P < 0.01, ***P < 0.001, ****P < 0.0001.

Analysis of CDK8 and CDK19 gene expression in 4,798 normal and 7,843 tumor tissue clinical samples from 16 different organs (Figure 1A) was conducted using RNA-Seq data in the TNMplot database (33). CDK19 is expressed at higher levels in androgen-dependent organs (prostate and testes) than in any other normal tissues. CDK19 expression further increases in PCa, reaching higher levels than in any other cancers; in contrast, CDK19 greatly decreases during testicular carcinogenesis. On the other hand, CDK8 is expressed at an intermediate level in the normal prostate and at a high level in the testes, but it is downregulated in both PCa and testicular cancers relative to their normal tissue counterparts (Figure 1A). Preferential elevation of CDK19 in PCa cells was also observed among the cell lines in the DepMap database. Among the top 1% of cell lines with the highest CDK19 expression (14 of 1,450), four (29%) belonged to the prostate lineage that constitutes less than 1% of all cell lines (12 of 1,450). Elevated CDK19 expression was associated with those prostate adenocarcinoma cell lines that express AR at a high level (Figure 1B). CDK19 elevation in AR-positive prostate cancers was confirmed at the protein level by Western blot analysis (Figure 1C), which compares CDK8, CDK19, and AR (including full-length AR [AR-FL] and AR variants [AR-Vs]) in different PCa cell lines (HEK293 [293] cells and 293 with knockout of both CDK8 and CDK19 [293-dKO] [ref. 34] were used as reference controls). CDK19 protein was strongly increased in AR-positive but not in AR-negative PCa cells (Figure 1C).

We analyzed the effects of androgen signaling on CDK8 and CDK19 RNA expression in androgen-responsive LNCaP cells. Cells were androgen deprived using medium with charcoal-stripped serum (CSS) for 48 hours, followed by the addition of R1881 androgen at 0.1, 1, and 10 nM for 24 hours. Quantitative PCR (qPCR) analysis (Figure 1D) showed that the expression of KLK3 (PSA), driven by canonical AR signaling, was abrogated by androgen depletion but induced by androgen addition in a concentration-dependent manner. CDK19 expression was unaffected by androgen depletion but was upregulated by androgen addition. In contrast, CDK8 expression was increased by androgen depletion but decreased to the basal level by androgen addition (Figure 1D). Hence, androgen signaling positively regulates CDK19 and negatively regulates CDK8 expression.

We compared the expression of markers of androgen signaling and Mediator kinase module subunits using RNA-Seq data from The Cancer Genome Atlas (TCGA) and cBioPortal databases for normal prostate tissues and primary (35) and metastatic PCa (the latter samples come from patients who failed ADT and therefore can be classified as mCRPC; ref. 36). The expression of AR and KLK3 (PSA) was elevated in primary tumors relative to normal prostate tissues, reflecting a carcinogenesis-associated increase in canonical AR signaling, whereas mCRPC showed a strong increase in AR but a decrease in KLK3 relative to primary tumors, indicating debilitation of canonical AR-driven transcriptional signaling (Figure 1E). CDK8 was downregulated in primary tumors, which is consistent with its negative regulation by androgen in LNCaP cells; however, CDK8 was strongly upregulated in mCRPC. In contrast, CDK19, which is positively regulated by androgen signaling, was strongly increased in primary PCa and further increased in mCRPC (the increase of CDK19 from primary PCa to mCRPC did not reach statistical significance, but it was previously documented in other studies; refs. 28, 37). The expression of all other Mediator kinase module components, CCNC, MED12, MED13, and MED13L (MED12L isoform is expressed at a very low level in PCa), increased from normal to primary to metastatic PCa (although the increases from normal to primary for MED13 and from primary to metastatic for MED12 did not reach statistical significance) (Figure 1E). We determined pairwise Pearson’s correlation coefficients between the expression of CDK8, CDK19, and KLK3 (used as a marker of canonical androgen signaling) in normal, primary, and metastatic prostate samples (Figure 1F). In agreement with the effects of androgen in LNCaP cells (Figure 1D), KLK3 expression was positively correlated with CDK19 and negatively correlated with CDK8 expression in all 3 sets of samples (Figure 1F).

These results indicate that downregulation of CDK8 and upregulation of CDK19 in primary PCa stem from the differential effects of androgen signaling on the expression of these genes and that the switch from canonical to non-canonical AR signaling in CRPC abrogates the negative regulation of CDK8 expression, allowing an increase in both Mediator kinases and their interactive proteins in mCRPC. These findings prompted us to investigate the role of Mediator kinases in the CRPC phenotype.

### Functional similarity of CDK8 and CDK19 in CRPC cells.

**Figure 2:**
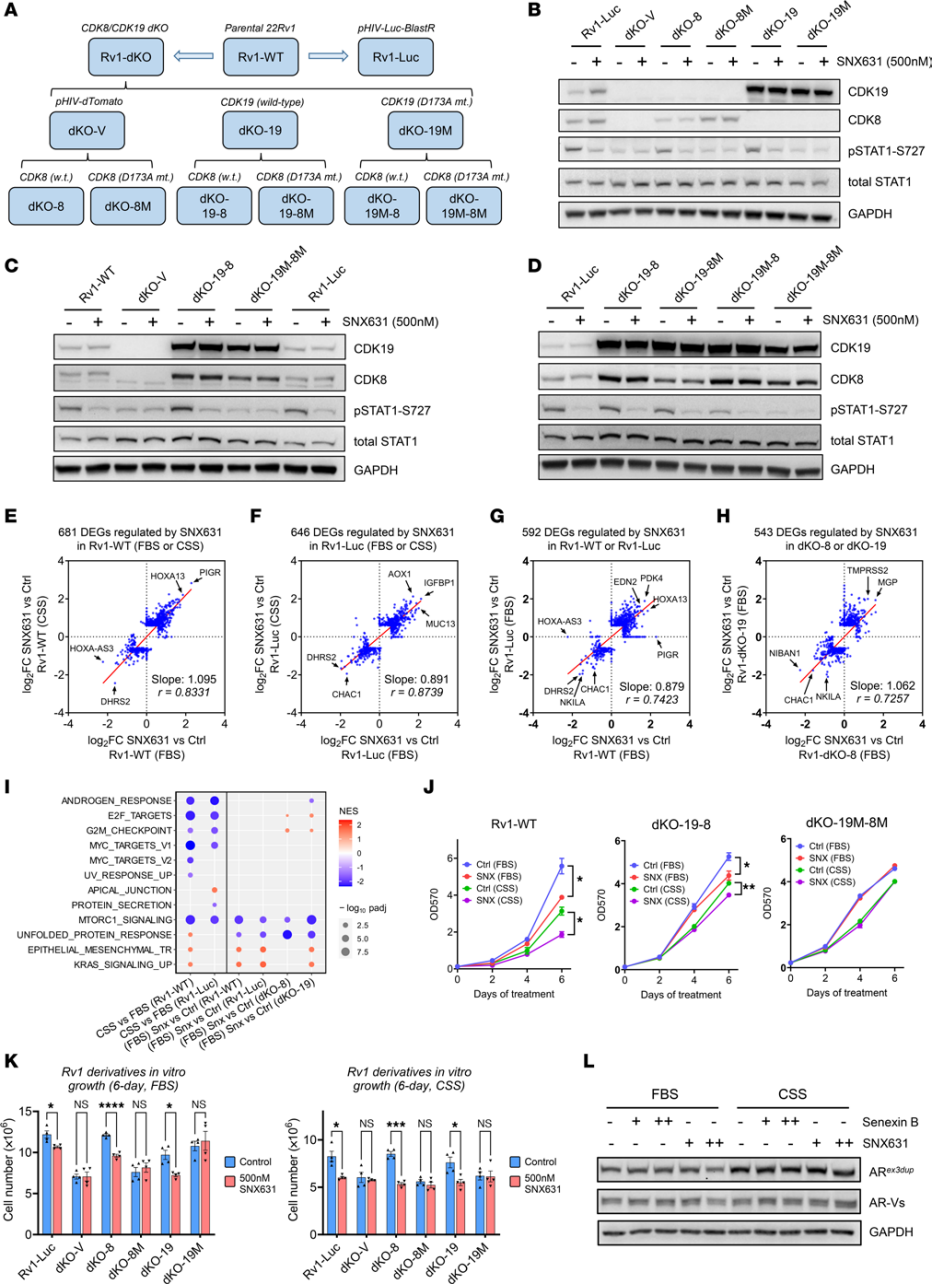
Effects of MKI in 22Rv1 derivatives in vitro. (**A**) Scheme of generation of CDK8/19-modified 22Rv1 derivatives. (**B**–**D**) Western blot analysis of the indicated proteins in 22Rv1 derivatives, untreated or treated with 500 nM SNX631 for 6 hours. (**E** and **F**) Comparison of the effects of Mediator kinase inhibition (MKI) on SNX631-affected DEGs between androgen-containing (FBS) and androgen-deprived (CSS) conditions in Rv1-WT (**E**) and Rv1-Luc (**F**) cells. (**G** and **H**) Comparison of the effects of MKI on SNX631-affected DEGs in Rv1-WT versus Rv1-Luc (**G**) and dKO-8 versus dKO-19 (**H**) in FBS medium. (**I**) Effects of MKI and androgen depletion on the affected hallmark pathways (GSEA) in 22Rv1 derivatives. (**J**) Effects of 500 nM SNX631 treatment (SNX) on 6-day growth curves of Rv1-WT, dKO-19-8, and dKO-19M-8M cells in FBS or CSS medium. (**K**) Effects of SNX631 on the cell number of 22Rv1 derivatives after 6-day growth in FBS or CSS medium. (**L**) Western blot analysis of the effects of 24-hour CDK8/19i treatment (1 μM Senexin B or 500 nM SNX631) on AR in 22Rv1 cells in FBS or CSS medium. *P < 0.05, **P < 0.01, ***P < 0.001, ****P < 0.0001.

To study the role of Mediator kinases in CRPC, we used 22Rv1 cells (38), a widely used CRPC model that expresses both a mutated version of AR-FL (AR*^ex3dup^*) and multiple C-terminally truncated AR variants (39, 40). To address the inherent phenotypic variability of 22Rv1 cells, we transduced parental 22Rv1 (Rv1-WT) with a lentivirus expressing firefly luciferase, yielding the derivative Rv1-Luc, which was subsequently compared with Rv1-WT in different assays. The role of Mediator kinases in 22Rv1 was analyzed using genetic modifications of CDK19 and CDK8, schematized in Figure 2A. 22Rv1 cells with a double knockout of CDK8 and CDK19 (Rv1-dKO) via CRISPR/Cas9 were described previously (8). We now generated Rv1-dKO derivatives that expressed wild-type CDK19 (dKO-19), its kinase-inactive D173A mutant (41) (dKO-19M), or an insert-free lentiviral vector (dKO-V), as well as dKO-V derivatives expressing wild-type CDK8 (dKO-8) or its kinase-inactive D173A mutant (dKO-8M) (42). At the next step, dKO-19 and dKO-19M derivatives were transduced with lentiviruses expressing wild-type CDK8 or its D173A mutant, generating cell lines designated dKO-19-8, dKO-19-8M, dKO-19M-8, and dKO-19M-8M (Figure 2A).

Figure 2, B–D, depicts the Western blot analysis of CDK8 and CDK19 expression in different derivatives and the effects of 6-hour treatment with selective CDK8/19i SNX631 on S727 phosphorylation of STAT1, a known substrate of Mediator kinases (43). Parental 22Rv1 cells express 4.5 times more CDK19 than CDK8 at the protein level (8). Remarkably, while dKO-19 and dKO-19M expressed a much higher level of CDK19 relative to parental cells, CDK8 expression in dKO-8 was even lower than in the parental cells (and lentiviral transduction efficiency of CDK8 into dKO cells was low relative to that of CDK19) (Figure 2B). In contrast, transduction of CDK8 into dKO-19 or dKO-19M cells was more efficient and allowed us to achieve a higher level of CDK8 expression than in the parental cells (Figure 2, C and D), suggesting that CDK8 protein may have been stabilized in the presence of CDK19. SNX631 treatment decreased STAT1 S727 phosphorylation in all the derivatives expressing wild-type CDK8 and/or CDK19, but not in dKO-V or in cells expressing only mutant Mediator kinases (Figure 2, B–D), confirming that the mutations abrogated kinase activity of both CDK8 and CDK19.

RNA-Seq was used to analyze the effects of SNX631 on gene expression in Rv1-WT and Rv1-Luc in androgen-containing medium (with fetal bovine serum [FBS]) and in androgen-depleted medium (with CSS) and in dKO-19, dKO-19M, dKO-8, and dKO-8M derivatives (in FBS). Differentially expressed genes (DEGs) affected by SNX631 in any derivative or culture condition were defined using fold change (FC) > 1.5 and false discovery rate (FDR) < 0.01 as cutoffs. These DEGs are listed in Supplemental Table 1 (supplemental material available online with this article; https://doi.org/10.1172/JCI176709DS1). Volcano plots in Supplemental Figure 1, A and B, show that growth in androgen-depleted medium (CSS vs. FBS) affected an order of magnitude more DEGs in Rv1-WT than in RV1-Luc cells, indicating that Rv1-Luc were less androgen responsive, reflecting the inherent heterogeneity of androgen sensitivity in PCa cell lines (3, 44). On the other hand, SNX631 affected similar numbers of DEGs of both cell lines in FBS or CSS (Supplemental Figure 1, A and B). Correlation analysis confirmed that the transcriptomic effects of SNX631 in FBS and CSS were similar in Rv1-WT (Figure 2E) and in Rv1-Luc (Figure 2F) as well as between the two 22Rv1 sublines (Figure 2G) despite a few differentially responding genes that may reflect clonal variations. SNX631 affected very few genes in Mediator kinase–mutated dKO-8M and dKO-19M derivatives, confirming the target selectivity of this CDK8/19i (Supplemental Figure 1, C and D).

SNX631 had similar qualitative and quantitative effects on gene expression in dKO-8 and dKO-19 cells, as indicated by the high correlation (*r* = 0.73) between the DEG expression levels and the slope close to 1 (1.06) (Figure 2H). Supplemental Figure 1E shows the effects of SNX631 on the expression of representative genes that are either positively or negatively regulated by Mediator kinases in the derivatives analyzed by RNA-Seq, as well as in Rv1-Luc and dKO-19-8, dKO-19-8M, dKO-19M-8, and dKO-19M-8M derivatives, where the expression of the same genes was analyzed by qPCR. Gene expression was unaffected by SNX631 in all the Mediator kinase–deficient derivatives but was responsive to CDK8/19i in all the derivatives expressing one or both wild-type Mediator kinases, confirming that CDK8 and CDK19 have similar effects on gene expression. The weakest response among the derivatives expressing active Mediator kinases was observed in dKO-19M-8 (Supplemental Figure 1E), which also showed the lowest p-STAT1 S727 expression (Figure 2D), suggesting that mutant CDK19 partially inhibited CDK8 activity.

Supplemental Figure 1F shows the heatmap of 33 genes that were regulated in vitro by MKI across all 22Rv1 derivatives analyzed by RNA-Seq. Gene set enrichment analysis (GSEA) (45) of 50 hallmark pathways revealed that androgen deprivation (CSS relative to FBS) downregulated the androgen response pathway as well as cell proliferation–related pathways (MYC, E2F, and G_2_/M) in Rv1-WT and Rv1-Luc cells (Figure 2I). Treatment with SNX631 significantly downregulated the unfolded protein response and mTORC1 pathways but had little or no effect on the androgen response pathway (Figure 2I), indicating that MKI has no major effect on AR signaling in CRPC cells.

The effects of MKI on in vitro growth of 22Rv1 derivatives were tested in androgen-containing (FBS) and in androgen-deprived (CSS) media in a 6-day assay. SNX631 treatment produced a moderate but significant inhibition of cell proliferation in both FBS and CSS in 22Rv1 derivatives expressing wild-type CDK8 and/or CDK19 (Rv1-WT, Rv1-Luc, dKO-8, dKO-19, dKO-19-8) but not in Mediator kinase–inactive dKO-V, dKO-8M, dKO-19M, or dKO-19M-8M cells (Figure 2, J and K). AR was unaffected by CDK8/19 inhibition at the RNA (Supplemental Figure 1G) or protein level (Figure 2L). Hence, CDK8 and CDK19 exert a moderate positive effect on 22Rv1 cell growth in vitro, irrespective of androgen supplementation and without altering AR expression or AR pathway activity.

### Mediator kinase inactivation reverses the castration-resistant phenotype of 22Rv1 CRPC xenografts in vivo.

**Figure 3:**
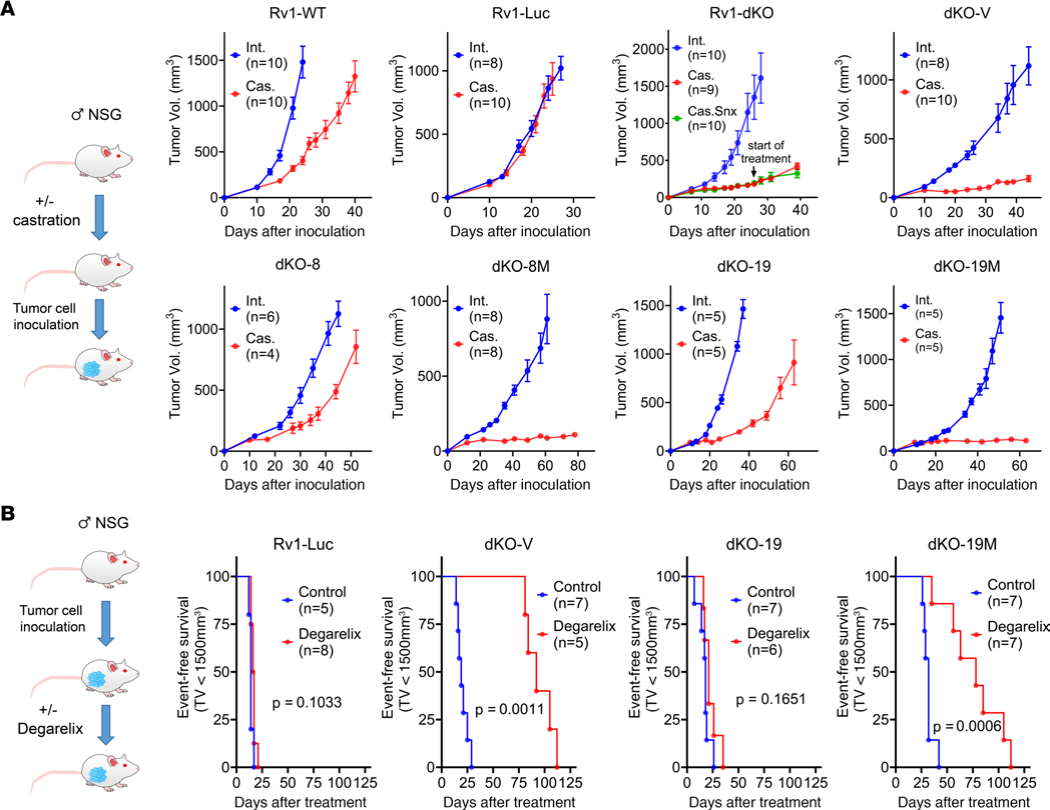
Effects of Mediator kinase mutagenesis on in vivo growth of 22Rv1 xenografts. (**A**) Scheme of the study and xenograft growth curves of the indicated 22Rv1 derivatives in intact and castrated NSG mice. Castration was performed surgically for RV1-WT and Rv1-dKO and chemically (10 mg/kg degarelix, s.c., every 30 days) for Rv1-Luc and dKO derivative studies. The Rv1-dKO study included an arm in which mice were treated with SNX631 (in medicated food at 500 ppm) starting on day 26 after implantation. (**B**) Scheme of the study and Kaplan-Meier plots of the effects of degarelix on event-free survival of NSG mice bearing the indicated 22Rv1 derivatives (event defined as a tumor volume exceeding 1.5 cm^3^).

We investigated the effects of CDK8 and CDK19 expression and kinase activity on in vivo growth of 22Rv1 xenografts, with or without androgen deprivation, by monitoring tumor growth of 22Rv1 derivatives in intact or castrated male NSG mice (Figure 3A). Rv1-WT and Rv1-Luc xenografts grew in both intact and castrated mice; castration slowed down the tumor growth for Rv1-WT but not for Rv1-Luc, in agreement with the lesser effect of androgen deprivation on gene expression in Rv1-Luc (Supplemental Figure 1, A and B). The CDK8/19-knockout derivatives Rv1-dKO and dKO-V grew in intact mice, but their growth in castrated mice was strongly suppressed (Figure 3A). Re-expression of CDK8 or CDK19 reproduced the phenotype of the Rv1-WT parent, as they grew in both intact and, at a slower rate, in castrated mice. dKO-19M and dKO-8M derivatives expressing inactive Mediator kinase mutants grew more slowly than the derivatives expressing the corresponding wild-type Mediator kinases in intact mice, and they formed measurable tumors but did not grow in castrated mice (Figure 3A). These results indicate that Mediator kinase activity supports in vivo growth of 22Rv1 xenografts, and that the effects of Mediator kinase inactivation become especially prominent under conditions of androgen deprivation.

To assess how Mediator kinase activity affects the response of established 22Rv1 tumors to ADT, we inoculated intact male mice with Rv1-Luc, dKO-V, dKO-19, and dKO-19M derivatives. When tumors reached 150–200 mm^3^ in size, mice were either untreated or treated with degarelix, a gonadotropin-releasing hormone antagonist that suppresses testosterone production in the body. The effect of degarelix on tumor growth was measured using event-free survival analysis, with the event defined as tumors reaching 1.5 cm^3^ volume. Degarelix treatment did not slow down the growth of Rv1-Luc or dKO-19 tumors that expressed functional Mediator kinases but drastically inhibited the growth of dKO-V and dKO-19M derivatives lacking Mediator kinase activity (Figure 3B). Hence, Mediator kinase inactivation restores the response to androgen deprivation in 22Rv1 CRPC tumors.

### Systemic CDK8/19i treatment suppresses androgen-independent in vivo growth and produces tumor regression and cures in 22Rv1 xenografts.

**Figure 4:**
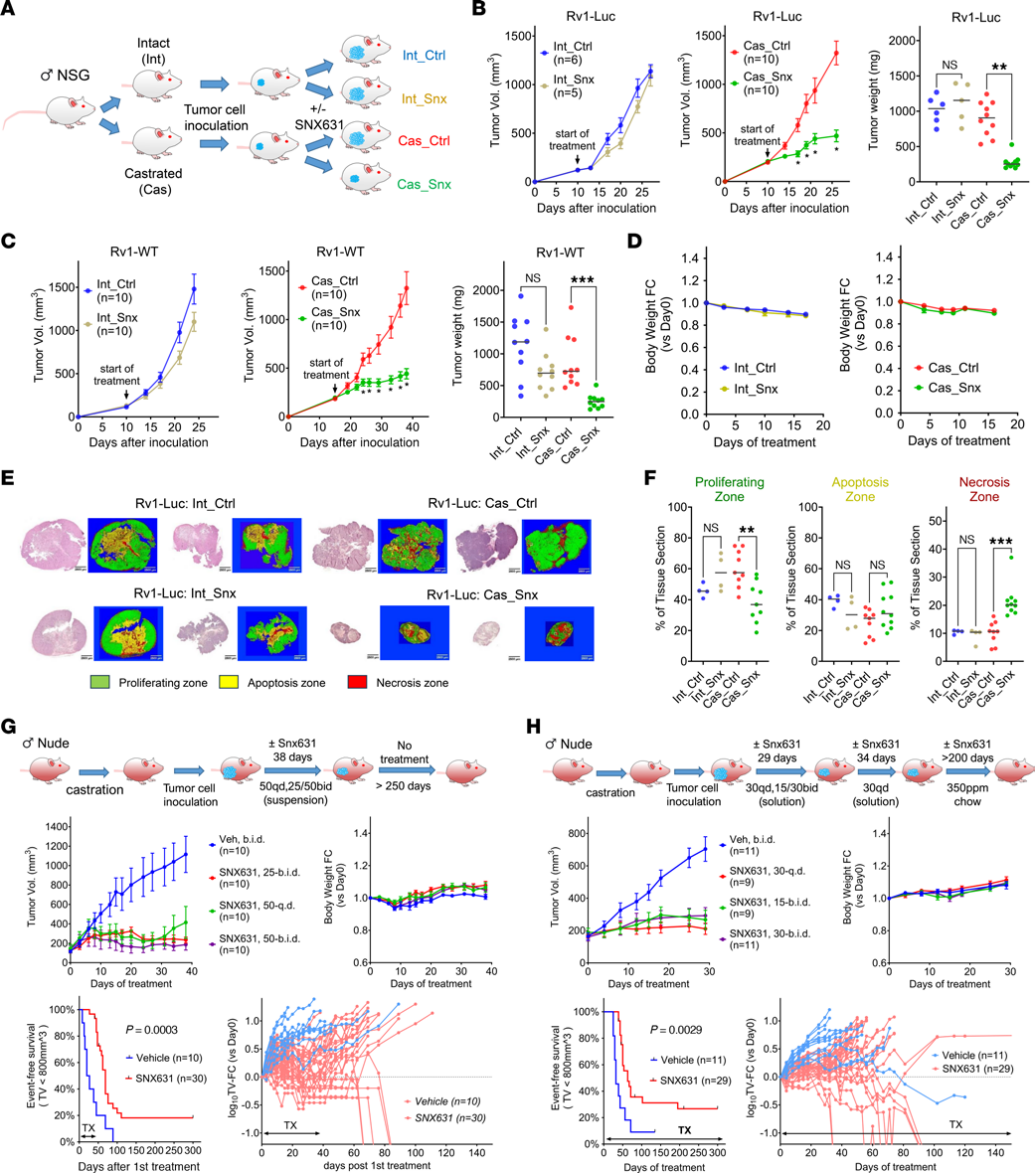
Effects of CDK8/19i on in vivo growth of 22Rv1 xenografts. (**A**) Scheme of the study. (**B**) Tumor growth curves and final tumor weights of Rv1-Luc xenografts growing in intact or castrated mice receiving the control or SNX631-medicated diet (500 ppm). (**C**) Tumor growth curves and final tumor weights of Rv1-WT xenografts grown in intact or castrated mice treated with SNX631 (30 mg/kg, bid) or vehicle via oral gavage. (**D**) Effects of SNX631 treatment (30 mg/kg, bid) on body weight changes for the animals in the studies in **C**. (**E**) Representative images of H&E staining and machine learning–based coloring of tumor sections from studies in **B**. Green, proliferation zone; yellow, apoptotic zone; red, necrotic zone. Scale bars: 2,500 μm. (**F**) Quantitation of the area of the indicated zones in tumor sections. (**G**) Castrated NCr nude mice were treated with the suspension vehicle or SNX631 (in suspension vehicle) at 25 mg/kg bid, 50 mg/kg qd, or 50 mg/kg bid for 39 days. Tumor volumes were monitored for 300 days from the start of treatment. Top: Tumor growth curves (left) and changes in mouse body weight (right). Bottom: Kaplan-Meier plot of event-free survival (left) and tumor growth in individual animals (right). (**H**) Castrated NCr nude mice were treated with solution vehicle or SNX631 (in solution vehicle) at 15 mg/kg bid, 30 mg/kg qd, or 30 mg/kg bid for 63 days and then treated with control or SNX631-medicated diet (350 ppm) for up to 300 days after the start of treatment; the plots are the same as for **G**. *P < 0.05, **P < 0.01, ***P < 0.001.

The effects of systemic in vivo treatment with CDK8/19i SNX631 on 22Rv1 tumors growing in intact or castrated male NSG mice were analyzed as shown in Figure 4A. Similarly to Mediator kinase mutagenesis, SNX631 had little effect on Rv1-Luc (Figure 4B) or Rv1-WT (Figure 4C) xenograft growth in intact mice but strongly inhibited the growth of both cell lines in castrated mice, as indicated by the effects on tumor volumes and final tumor weights (Figure 4, B and C). SNX631 had no detrimental effect on body weight in intact or castrated mice compared with vehicle groups (Figure 4D). Machine learning–based histological analysis indicated that tumor suppression by SNX631 in castrated mice was associated with decreased cell proliferation and increased necrosis (Figure 4, E and F). SNX631 had no effect on the growth of CDK8/19-deficient Rv1-dKO tumors in castrated mice (Figure 3A), confirming that the effect of the inhibitor was mediated by CDK8/19.

We investigated the long-term effects of systemic treatment with SNX631 in Rv1-WT xenografts growing in castrated NCr nude mice, which, unlike NSG, contain NK cells that are known to be stimulated by CDK8/19i (20, 21). In the first study (Figure 4G), mice were treated with SNX631 for 38 days. CDK8/19i slowed down tumor growth relative to the control group, with no detrimental effect on mouse body weights. Remarkably, some of the treated tumors stopped growing and even showed regression. After treatment, mice continued to be monitored for 300 days with tumor size measurements. The majority of tumors resumed growth after cessation of treatment, but a notable subset (5 of 30, 16.7%) continued to regress and eventually disappeared (Figure 4G), indicating the achievement of cures. In the second study (Figure 4H), SNX631 treatment was continued for the entire 300-day period. No detectable adverse effects were observed during the entire treatment period (about half of the mouse lifespan), although a few mice (1 of 11 in the vehicle group and 3 of 29 in the SNX631 group) died for treatment-unrelated reasons. Complete tumor disappearance without recurrence was observed in 25% of animals receiving continuous CDK8/19i treatment.

### Cooperative transcriptomic effects of MKI and castration in tumor cells.

To understand why MKI strongly inhibited CRPC growth in vivo in castrated mice but had weaker effects in intact animals or in vitro, we performed RNA-Seq analysis of tumors formed in intact and castrated NSG mice in 3 different 22Rv1 models of MKI: Rv1-WT and Rv1-Luc, treated or untreated with SNX631, and 22Rv1 derivatives expressing wild-type (dKO-19) or mutant (dKO-19M) CDK19. RNA-Seq data were analyzed separately for human (tumor) and stromal (mouse) RNA, as described previously (46). The numbers of tumor-derived DEGs obtained in different comparisons using FDR < 0.01 and FC > 1.5 as cutoff criteria are shown in the volcano plots in Supplemental Figure 2, A–C.

**Figure 5:**
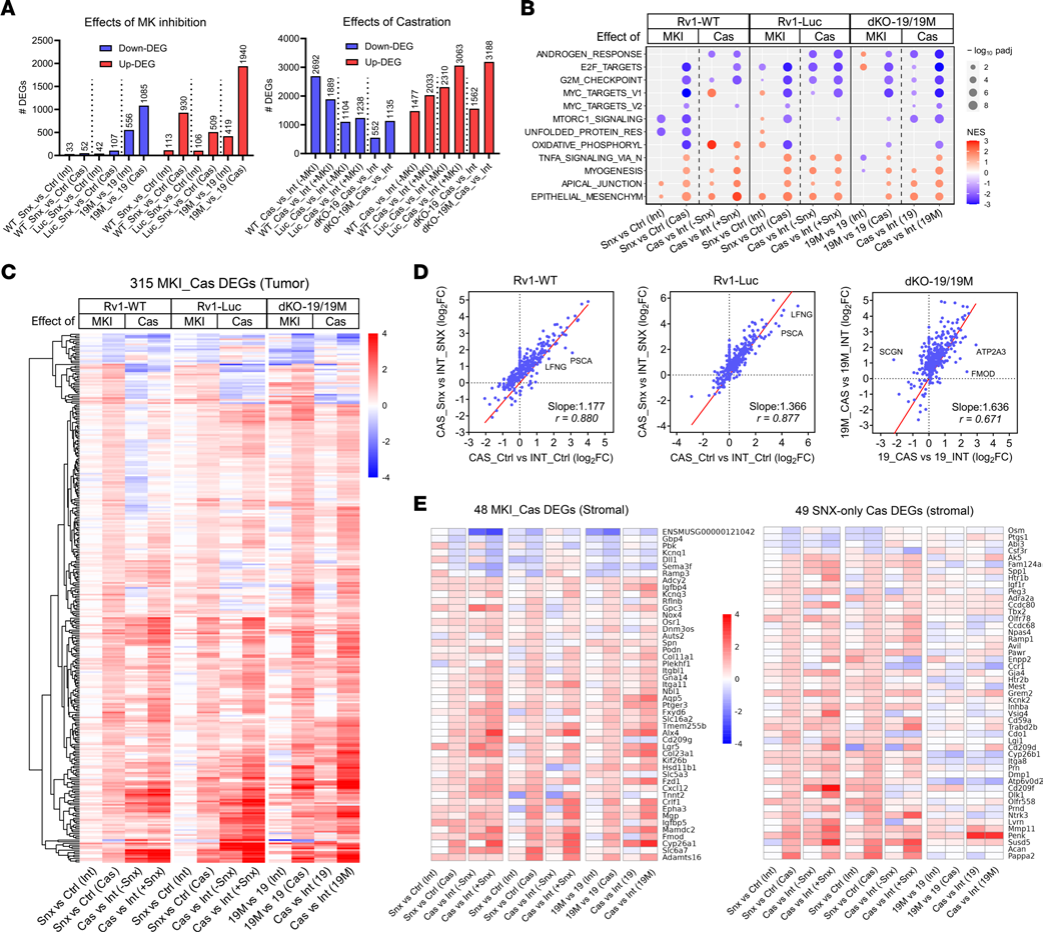
Transcriptomic effects of MKI on tumor (human) and stromal (mouse) genes in 22Rv1 xenografts. (**A**) Number of tumor DEGs affected by MKI in Rv1-WT and Rv1-Luc (treated or untreated with SNX631) and in cells expressing wild-type (dKO-19) or kinase-deficient (dKO-19M) CDK19, in tumors growing in castrated (Cas) or intact (Int) male mice. (**B**) Effects of MKI and castration under the indicated conditions on the affected hallmark pathways in three 22Rv1 tumor models. (**C**) Heatmap of 315 tumor DEGs coregulated by MKI in three 22Rv1 models in the indicated comparisons. (**D**) Correlation between the effects of castration with and without MKI on the same DEGs in three 22Rv1 models. (**E**) Heatmaps of stromal DEGs affected in castrated animals by both SNX631 treatment and Mediator kinase mutagenesis (left) or by SNX631 treatment but not by Mediator kinase mutagenesis (right) under the indicated conditions.

Castration had a major effect on tumor gene expression (both upregulation and downregulation) even when it did not suppress tumor growth (as in Rv1-Luc) (Supplemental Figure 2, A–C, and Figure 5A). Fewer genes were affected by MKI, and in almost all cases (except for dKO-19M vs. dKO-19 in intact mice), MKI induced many more genes than it inhibited. Remarkably, the number of MKI-induced genes was much greater (4.6- to 8.2-fold) in tumors growing in castrated than in intact mice (Figure 5A and Supplemental Figure 2, A–C). The majority of MKI-responsive DEGs in tumors growing in castrated animals were not affected in intact mice or in vitro (Supplemental Figure 2D). The much broader transcriptomic effects of MKI in tumors growing in castrated mice concur with the preferential suppression of such tumors by MKI. Remarkably, a high fraction of the DEGs affected by MKI in castrated mice were also affected by castration (from 27% to 56% in different 22Rv1 models) (Supplemental Figure 2E). Furthermore, the number of DEGs upregulated by castration increased 1.3- to 2.0-fold under MKI (Figure 5A and Supplemental Figure 2, A–C), suggesting that Mediator kinase activity restrains many castration-induced transcriptomic changes.

GSEA showed that MKI affected 12 of the 50 hallmark pathways (Figure 5B), but the androgen response pathway was affected in only 1 of 3 models. Several pathways were downregulated in all 3 models in castrated but not in intact mice, including those related to cell proliferation (MYC and E2F targets, G_2_/M checkpoint) and the mTORC1 signaling and oxidative phosphorylation pathways. Several other pathways (TNFA signaling, myogenesis, apical junction, epithelial-mesenchymal transition) were upregulated by MKI in castrated but not in intact mice, and such pathways were preferentially enhanced by castration under the conditions of MKI (Figure 5B). The latter pattern was especially noticeable among the transcription factor pathways (from the C3 transcription factor targets legacy collection), where the effect of castration was greatly increased by MKI (Supplemental Figure 2F), providing a further indication that Mediator kinase activity restrained the transcriptional effects of castration.

To identify genes that may be involved in tumor suppression in all three 22Rv1 models in castrated mice, we selected 315 DEGs that were coregulated by SNX631 treatment in both Rv1-WT and Rv1-Luc tumors and differentially expressed between dKO-19M and dKO-19 tumors in castrated mice (Supplemental Table 2). The effects of MKI or castration on the expression of these genes are shown in the heatmap in Figure 5C. Only 20 (6.3%) of these DEGs were downregulated by MKI, whereas the rest were upregulated. The effects of MKI in castrated mice and the effects of castration under the conditions of MKI on these 315 DEGs were strikingly similar (Figure 5C). There was a significant correlation between the effects of castration and MKI in castrated mice on these DEGs (Supplemental Figure 3A). Castration had a markedly stronger effect on these genes under the conditions of MKI (Figure 5D), as indicated by slope greater than 1. This suggests that MKI largely enhanced the transcriptomic effects of castration in tumor cells, although a few genes (such as PSCA or SCGN) showed opposite responses to castration and MKI (Supplemental Figure 3A). The hyperactivation of castration-inducible genes by MKI resembles the hyperinduction of super-enhancer–associated genes by CDK8/19 inhibition in AML cells (13).

Supplemental Figure 4, A and B, shows the effects of different treatments in vitro and in vivo on the expression of selected DEGs that represent distinct patterns of response to castration and MKI. Among the genes upregulated by castration but downregulated by MKI, we note PCa biomarkers PSCA (47) and FMOD (48), whereas genes such as SORD (an androgen-responsive gene; ref. 49) and ENSG00000289695 are downregulated both by castration and by MKI (Supplemental Figure 4A). In the much larger category of genes that are induced by castration and hyperinduced when castration is combined with MKI (Supplemental Figure 4B), we note an AR-regulated WNT protein, WNT7B (50), and the WNT inhibitor DKK1 (51), as well as annexin ANXA3 and LFNG, a gene implicated in tumor suppression in PCa (52). Remarkably, 12–14 keratin genes were strongly induced by a combination of castration and MKI (Supplemental Figure 5, A and B). Interestingly, ETV6 and FOSL2, 2 of 6 genes identified in AML as associated with super-enhancers, hyperactivated by MKI, and inhibiting cell proliferation when overexpressed (13), were also upregulated by Mediator kinase inactivation in 22Rv1 tumors in castrated mice (Supplemental Figure 4C).

Supplemental Figure 4D shows the effects of different treatments on the expression of MYC and some of the strongly affected MYC targets. The effects of castration on MYC expression matched its effects on tumor growth (Figure 3A), decreasing MYC expression in Rv1-WT and, to a lesser extent, in dKO-19, but not in castration-resistant Rv1-Luc. MKI had no significant effect on MYC expression but, when combined with castration, decreased the expression of MYC targets, such as MCM4, CCNA2, MAD2L1, CDK4, and SERBP1 (Supplemental Figure 4D), paralleling the effects of castration and MKI on tumor growth. This result suggested a possible role of the MYC pathway in the effects of MKI.

### Both systemic treatment with a CDK8/19i and Mediator kinase mutagenesis in tumor cells affect stromal gene expression.

We analyzed the effects of MKI on stroma-derived (mouse) genes in the three 22Rv1 tumor models. The number of mouse reads from these tumors was low relative to the number of human reads (Supplemental Figure 6A), and therefore our statistical analysis of stromal genes was limited. Nevertheless, we identified 97 stromal DEGs that were affected by SNX631 treatment of Rv1-WT and Rv1-Luc tumors in castrated mice (using *P* < 0.05 and FC > 1.5 as the cutoff criteria). Surprisingly, half of these genes (48 DEGs) were affected not only by systemic treatment with SNX631 but also by Mediator kinase mutation in tumor cells alone, suggesting that Mediator kinase activity in tumor cells is involved in shaping the tumor microenvironment. Heatmaps of the effects of different treatments on the DEGs affected both by Mediator kinase mutagenesis in tumor cells and by SNX631 treatment or only by systemic treatment with SNX631 are shown in Figure 5E. The effects of MKI and castration on stromal gene sets were correlated in 2 of the 3 models (Supplemental Figure 3B), and MKI increased the effects of castration on these genes (slope >1) (Supplemental Figure 3C). The expression of selected stromal genes representing different regulatory patterns is shown in Supplemental Figure 6, B and C. We note that changes in some of the stromal genes could have contributed to the inhibition of tumor growth in castrated mice, such as upregulation of Igfbp4 (53), Ccdc80 (54), and Dlk1 (55) and downregulation of Ramp3 (56) and Osm (57).

### MKI suppresses in vivo growth of a MYC-driven transgenic CRPC model.

Since the MYC pathway, a major driver of advanced PCa, was one of the pathways selectively downregulated by MKI in 22Rv1 xenografts growing in castrated mice, we asked whether CDK8/19i treatment would affect the growth of MYC-CaP-CR (58), a derivative of the MYC-driven transgenic PCa model MYC-CaP (59) selected for the ability to grow in castrated mice. As a CDK8/19i, we used here SNX631-6, an equipotent analog of SNX631. Supplemental Figure 7 shows the structure of SNX631-6 (Supplemental Figure 7A), its CDK8/19 selectivity based on kinome profiling (Supplemental Figure 7B), its potency in a CDK8/19-dependent cell-based assay (Supplemental Figure 7C), and the comparison of the CDK8/19i Senexin B (15), SNX631, SNX631-6, and the nonselective kinase inhibitor staurosporine in regard to cell-free binding kinetics for CDK8 and CDK19 (Supplemental Figure 7D).

**Figure 6:**
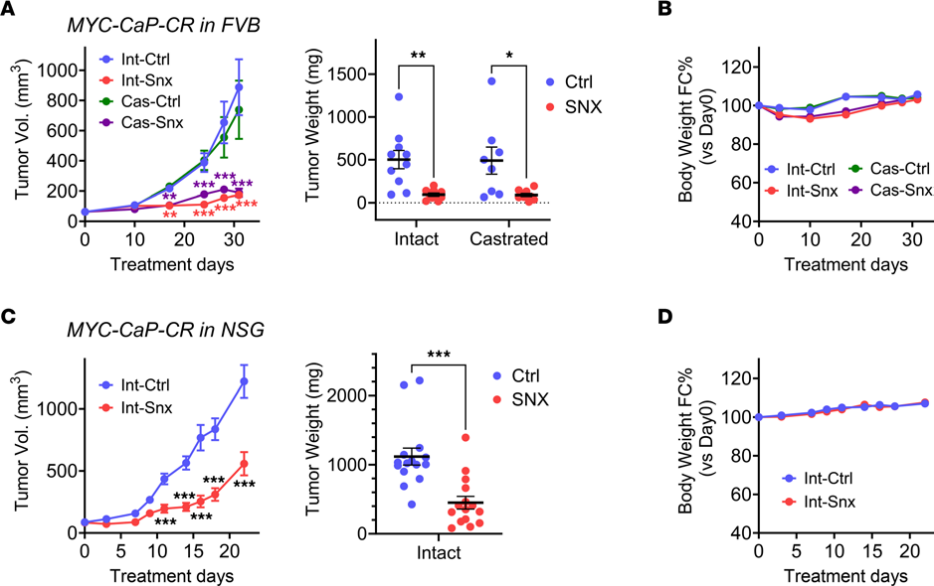
Effects of CDK8/19i on MYC-CaP-CR in vivo growth. (**A**) Tumor growth curves and final tumor weights of MYC-CaP-CR tumors growing in intact or castrated FVB mice receiving the control or SNX631-6–medicated diet (500 ppm). (**B**) Effects of SNX631-6 treatment on body weight changes of mice in the studies in **A**. (**C**) Tumor growth curves and final tumor weights of MYC-CaP-CR tumors growing in intact NSG mice receiving the control or SNX631-6–medicated diet (500 ppm). (**D**) Effects of SNX631-6 treatment on body weight changes of mice in the studies in **C**. *P < 0.05, **P < 0.01, ***P < 0.001.

The growth of MYC-CaP-CR tumors in isogenic FVB mice was unaffected by castration, but it was almost completely inhibited by systemic treatment with SNX631-6, in both castrated and intact animals (Figure 6A). SNX631-6 treatment had no detrimental effect on body weight (Figure 6B). Since MKI is known to stimulate the antitumor activity of both NK (20, 21) and effector T cells (22), we asked whether tumor suppression in this model could be due to the use of an immunocompetent host. To test this, we analyzed the effect of SNX631-6 on MYC-CaP-CR tumor growth in immunodeficient NSG mice. CDK8/19i suppressed tumor growth in NSG mice (Figure 6C), with no detrimental effects on body weight (Figure 6D), but MYC-CaP-CR tumor suppression was not as complete in NSG mice as in the immunocompetent FVB. These results indicate that the strong tumor-suppressive effect of the CDK8/19i on MYC-CaP-CR was not dependent on castration but was most likely mediated by downstream inhibition of MYC signaling and could possibly involve immune stimulation by CDK8/19i.

### MKI in androgen-dependent PCa suppresses PSA expression with little effect on cell proliferation.

**Figure 7:**
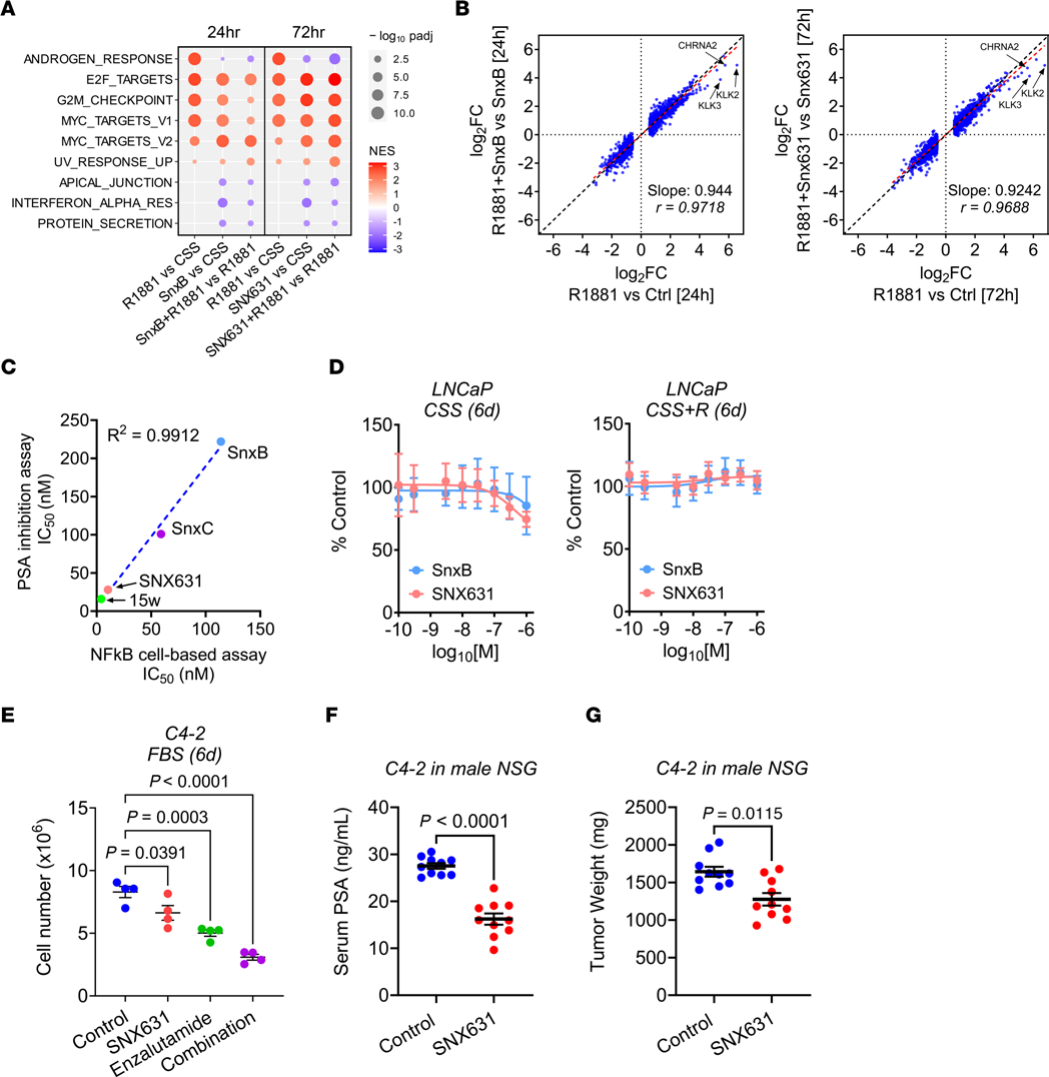
Effects of MKI in androgen-responsive PCa cells. (**A**) Hallmark pathways (RNA-Seq, GSEA) affected in LNCaP cells by R1881 androgen and CDK8/19i Senexin B (SnxB) or SNX631 in CSS medium. (**B**) Effects of treatment with 2 μM Senexin B or 500 nM SNX631 relative to the effects of R1881 on androgen-regulated genes in LNCaP cells. (**C**) Correlation of IC_50_ values of different CDK8/19i based on PSA ELISA in C4-2 cells and NF-κB reporter assay in 293 cells. (**D**) Effects of Senexin B and SNX631 on the growth of LNCaP cells in CSS medium with or without 100 pM R1881, measured by the sulforhodamine B assay. (**E**) Effects of SNX631 (500 nM) and enzalutamide (5 μM), individually or in combination, on the 6-day growth of C4-2 cells in FBS medium. (**F** and **G**) Serum PSA (**F**) and final tumor weights (**G**) of C4-2 xenografts grown in intact male NCG mice, treated with SNX631 (25 mg/kg, bid) or vehicle for 14 days.

Since CDK8 and CDK19 are regulated by androgen, we asked whether their inhibition would affect androgen signaling in androgen-responsive LNCaP cells, by conducting RNA-Seq analysis of androgen-dependent LNCaP cells, untreated or treated with 2 chemically unrelated CDK8/19i, Senexin B (used at 2 μM) and the more potent SNX631 (used at 0.5 μM), with or without R1881 androgen stimulation for 24 or 72 hours. Transcriptomic effects of androgen and CDK8/19i treatment are shown in the volcano plots in Supplemental Figure 8. DEGs selected using FDR < 0.01 and FC > 1.5 as cutoff criteria are listed in Supplemental Table 3. GSEA revealed that 9 of the 50 hallmark pathways were significantly affected by the CDK8/19i in LNCaP cells, with or without androgen stimulation (Figure 7A). Several pathways associated with cell proliferation (MYC and E2F targets; G_2_/M) and UV response were upregulated by androgen and further upregulated by the CDK8/19i in androgen-dependent LNCaP cells. On the other hand, the androgen response pathway was strongly stimulated by androgen but the addition of CDK8/19i reduced this response (in contrast to the lack of such effect in 22Rv1 CRPC cells). Figure 7B shows the effects of androgen (R1881) on androgen-regulated DEGs (2,364 DEGs for 24-hour and 2,579 DEGs for 72-hour treatments), in the presence and in the absence of CDK8/19i. Neither Senexin B nor SNX631 had a significant effect on most of the androgen-regulated DEGs, but both of them decreased androgen induction of the most strongly androgen-responsive genes, including CHRNA2, KLK2, and KLK3 (PSA) (Figure 7B).

The effects of CDK8/19i on the expression of PSA, the principal biomarker of PCa, were validated at the protein level by measurement of secreted PSA in the conditioned media from LNCaP and several other PCa cell lines (C4-2, LN3, VCaP, 22Rv1) treated with different concentrations of CDK8/19i Senexin B, SNX631, Senexin C (25), and 15w (60) (Supplemental Figure 8, B–D). The IC_50_ values for PSA inhibition perfectly correlated (*R*^2^ = 0.99) with the IC_50_ values for all three CDK8/19i in a cell-based assay (25, 34) (Figure 7C), confirming that the effect on PSA was mediated by CDK8/19.

To determine whether the transcriptomic effects of CDK8/19i are associated with an effect on proliferation of androgen-responsive cells in the absence or presence of androgen, we evaluated the effects of a 6-day treatment with different concentrations of Senexin B and SNX631 on LNCaP cell growth in CSS with or without the addition of R1881. The CDK8/19i mildly inhibited cell growth in androgen-deprived (CSS) medium but had no significant effect on cell number when androgen was added (Figure 7D). We also analyzed the effects of SNX631, alone and in combination with enzalutamide, on in vitro growth of LNCaP-derived C4-2 cells, which exhibit partial androgen independence but remain androgen responsive (61, 62). SNX631 moderately but significantly inhibited C4-2 cell proliferation and potentiated the effect of enzalutamide (Figure 7E). We further examined the in vivo effects of SNX631 treatment on tumor growth and serum PSA production by C4-2 xenografts in intact male NCG mice. After 11 days of treatment, CDK8/19i treatment strongly decreased serum PSA levels (Figure 7F) and moderately but significantly inhibited tumor growth, based on the final tumor weights (Figure 7G). C4-2 cells, however, showed a poor tumor take in castrated mice, and therefore the in vivo effects of MKI in this model could not be evaluated under the conditions of androgen deprivation.

### MKI suppresses patient-derived xenograft models of AR-expressing CRPC both in castrated and in intact mice.

**Figure 8:**
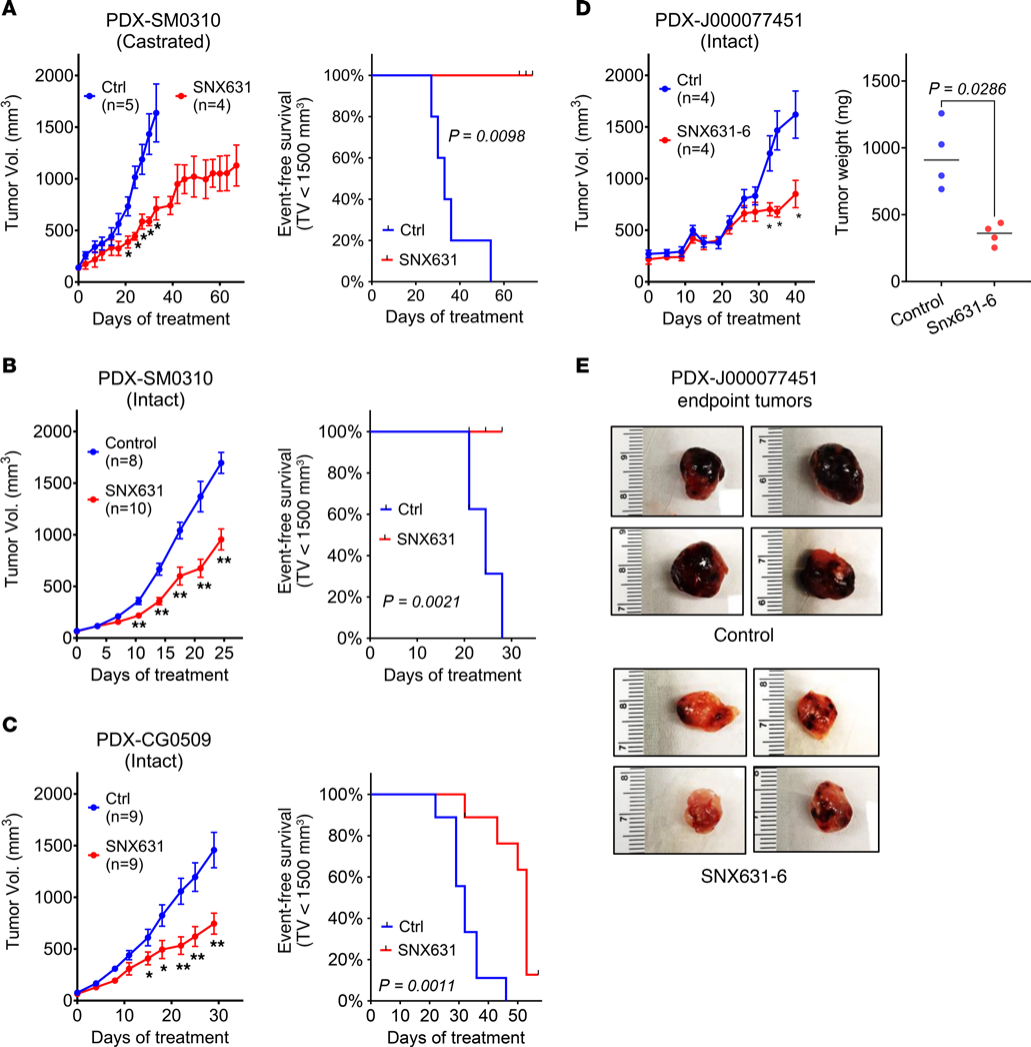
Effects of CDK8/19i on CRPC PDX growth in castrated and intact mice. (**A** and **B**) Effects of SNX631 treatment (medicated food, 500 ppm) on tumor growth and event-free survival of SM0310 PDX model in castrated (**A**) or intact (**B**) male NSG mice. (**C**) Effects of SNX631 treatment on tumor growth and event-free survival of CG0509 PDX model in intact NSG mice. (**D**) Effects of SNX631-6 treatment (medicated food, 500 ppm) on J000077451 PDX tumor growth in intact NSG mice. (**E**) Macroscopic images of control and SNX631-6–treated J000077451 PDX endpoint tumors. *P < 0.05, **P < 0.01.

We investigated the effects of CDK8/19i on the growth of 3 patient-derived xenograft (PDX) models of AR-positive CRPC. The first two, SM0310 and CG0509, were derived from prostate adenocarcinomas of patients who failed both ADT and chemotherapy (Supplemental Figure 9A) and displayed positive AR immunostaining (Supplemental Figure 9B). The third PDX, J000077451 (The Jackson Laboratory), was derived from the brain metastasis of grade IV prostate adenocarcinoma and expressed high levels of AR RNA. Although the patient’s treatment history is unknown, the growth of this PDX is resistant to cisplatin and is weakly inhibited by docetaxel (The Jackson Laboratory website). Only 1 of the 3 PDX models, SM0310, showed sufficient tumor take and could be tested for the response to CDK8/19 inhibition in castrated NSG mice. SNX631 treatment inhibited the tumor growth of SM0310 in both castrated (Figure 8A) and intact (Figure 8B) male NSG mice. Remarkably, prolonged treatment with SNX631 (67 days) stabilized PDX growth in castrated mice once the size of the xenograft reached about 1,000 mm^3^ (Figure 8A). CG0509 growth was tested only in intact mice, where it was nevertheless inhibited by SNX631 (Figure 8C).

Treatment with the CDK8/19i SNX631-6 did not affect J000077451 PDX growth in intact mice for the first 20–25 days of treatment, but strongly inhibited the tumor growth afterward (Figure 8D). Interestingly, the untreated J000077451 tumors displayed a high blood content, as indicated by a dark-colored hemorrhagic phenotype, but the SNX631-6–treated tumors were lighter in color and less hemorrhagic (Figure 8E). H&E staining showed that the control tumors contained cavities filled with blood cells, which were not observed in SNX631-6–treated tumors (Supplemental Figure 9C), suggesting that the delayed tumor-suppressive effect of Mediator kinase inhibitor in this model could be due to interference with tumor blood supply.

### Gene signature of Mediator kinase activity correlates with tumor progression and overall survival in mCRPC.

**Figure 9:**
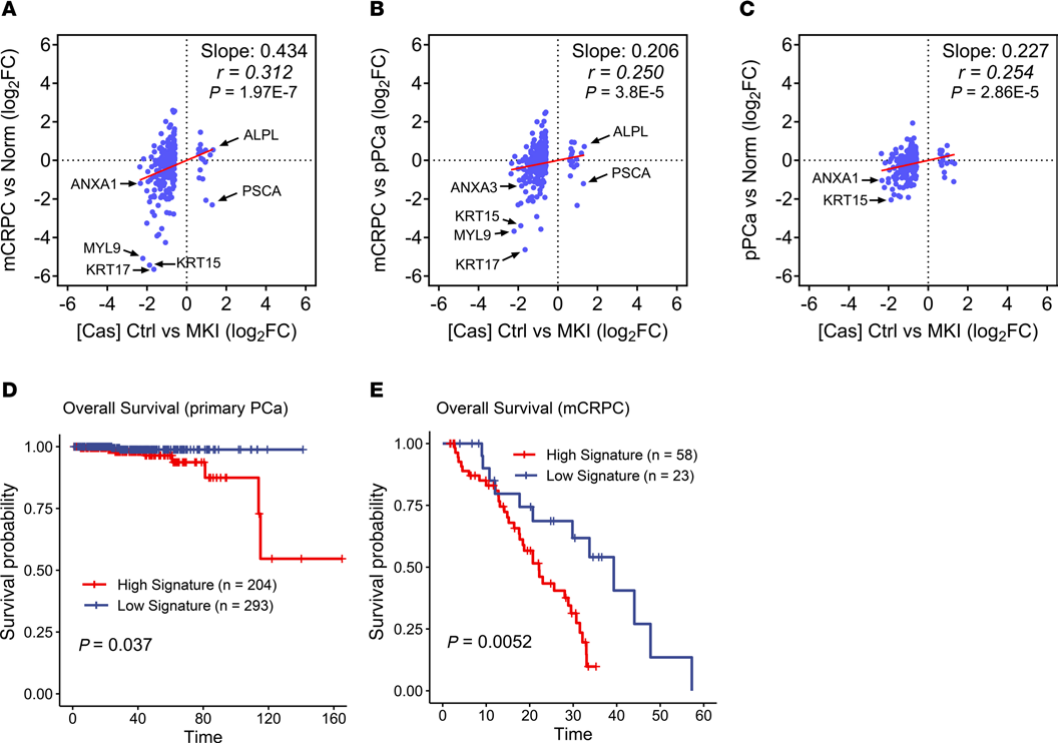
Expression and survival correlations in clinical PCa samples for genes affected by MKI in 22Rv1 CRPC xenografts. (**A**–**C**) Correlations of the differences in gene expression of 266 DEGs affected by MKI in 22Rv1 xenografts and expressed in clinical PCa between 22Rv1 tumors growing in castrated animals without and with MKI (average fold change for three 22Rv1 models) with the differences between clinical samples of mCRPC versus normal prostate (**A**), mCRPC versus primary PCa (**B**), and primary PCa versus normal prostate (**C**). (**D** and **E**) Correlations of the gene signature comprising the same 266 DEGs with overall survival in primary PCa (**D**) and mCRPC patients (**E**).

We asked whether the effects of MKI on gene expression in 22Rv1 xenografts correlate with differences in gene expression between mCRPC, primary PCa, and normal prostate tissues, using the same RNA-Seq clinical data sets as in Figure 1E. Among the 315 MKI-Cas DEGs identified from three 22Rv1 models (Figure 5C), 266 genes were expressed (fragments per kilobase per million mapped reads [FPKM] > 0.1) in mCRPC clinical samples and selected for the analysis (Supplemental Table 4). Differences in the expression of this set of genes in 22Rv1 tumors with or without MKI correlated with changes in their expression between mCRPC relative to normal prostate, mCRPC relative to primary PCa, and primary PCa relative to normal prostate tissues (Figure 9, A–C), indicating that the effects of CDK8/19 on gene expression are increased during prostate carcinogenesis and the progression of PCa to mCRPC. We then tested whether the same 266-gene Mediator kinase signature correlated with overall survival (OS) in the RNA-Seq data sets of 497 primary PCa and 81 mCRPC patients for whom survival data were available (36). The Mediator kinase activity signature showed a negative correlation with OS among primary PCa patients (Figure 9D) and an especially strong correlation among the patients with mCRPC (Figure 9E).

## Discussion

The incurability of advanced PCa is largely attributed to the remarkable plasticity of CRPC cells and their propensity for cellular reprogramming, which underlies such plasticity (3). Our findings indicate that Mediator kinase paralogs CDK19 and CDK8, which function as broad-spectrum regulators of transcriptional reprogramming (10), play the key role in the ability of CRPC tumors to grow under the conditions of androgen deprivation in vivo. As a result, the acquisition of androgen independence renders CRPC dependent on Mediator kinase activity. This dependence offers a novel therapeutic opportunity for suppressing CRPC growth and even producing cures in this presently incurable disease by treatment with Mediator kinase inhibitors, a new class of drugs in the clinical development pipeline.

The involvement of Mediator kinases in PCa likely reflects their inherent roles in prostate tissue physiology. The prostate and the other androgen-dependent organ, the testis, have the highest CDK19 expression among normal tissues; the testis also has the highest expression of CDK8. Our finding that androgen downregulates CDK8 and upregulates CDK19 in androgen-responsive PCa cells offers a rare example of the regulation of Mediator kinase expression by a physiological agent. The analysis of Mediator kinase expression in clinical cancers, together with prior reports (26), shows that CDK8 is downregulated and CDK19 upregulated in primary PCa, which we can now explain by the regulation of CDK8 and CDK19 expression by androgen signaling. (In contrast to the prostate, testicular carcinogenesis is associated with a uniquely strong downregulation of both CDK8 and CDK19. Perhaps not coincidentally, testicular carcinoma is the most chemotherapy-curable cancer in adults.) Downregulation of CDK8 ceases, however, when PCa progresses to CRPC and canonical androgen signaling is abrogated, at which stage CDK8 becomes strongly upregulated, along with continued increase in CDK19. As shown in a previous study, both CDK19 and CDK8 are upregulated in CRPC at the protein level (26). Our finding that exogenous CDK8 protein expression can be much higher in the cells that express CDK19 than in CDK19-deficient cells suggests that elevated CDK19 may augment CDK8 expression at the protein level. The other components of the kinase module of the Mediator complex (CCNC, MED12, MED13, MED13L) are also elevated in CRPC, suggesting that such upregulation is likely driven by selection for increased Mediator kinase activity.

Analysis of the transcriptomic effects of MKI on androgen-regulated transcription in androgen-responsive LNCaP cells showed that CDK8/19i both counteracted the induction of the most strongly androgen-responsive genes (including PSA) and enhanced the effect of androgen on a subset of genes. MKI did not inhibit the mitogenic effects of androgen on LNCaP cells in vitro but mildly reduced cell proliferation under androgen-deprived conditions. MKI also inhibited in vitro proliferation and potentiated the effect of enzalutamide in the LNCaP derivative C4-2, in agreement with a prior report on other androgen-responsive LNCaP derivatives (31).

To understand the role of Mediator kinases in CRPC, we analyzed their effects on transcription and tumor growth using both mutagenesis of CDK8 and CDK19 and pharmacological inhibition of Mediator kinases in 22Rv1, a “classical” androgen-independent CRPC model. The effects of CDK8 and CDK19 on gene expression and phosphorylation of a Mediator kinase substrate (STAT1 S727) were qualitatively similar in 22Rv1, in agreement with previous studies in other cell lines (8). While 22Rv1 proliferation in vitro was only moderately inhibited by MKI (regardless of the presence of androgen in cell culture), the tumor growth in vivo was dramatically suppressed by MKI in castrated mice but much less affected in intact male animals. Furthermore, CDK8/19 inactivation rendered tumors formed by these CRPC cells responsive to a conventional ADT agent (degarelix). Hence, Mediator kinase activity is required for CRPC growth in vivo under the conditions of androgen deprivation.

The mechanism underlying the selective effect of MKI on in vivo growth of 22Rv1 CRPC in castrated mice was investigated using RNA-Seq. This analysis revealed that MKI had a much greater effect on gene expression in tumors growing in castrated than in intact male mice (or in cell culture), with castration increasing the number of MKI-induced genes 4.6- to 8.2-fold. Of the tumor genes affected by MKI in castrated animals, 27%–56% were also affected by castration. Only a few genes showed opposite responses to castration and MKI. Remarkably, this small group included PSCA, a positive regulator of PCa growth and metastasis (63), which was upregulated by castration but downregulated by MKI. The small number of such genes stands in contrast to other systems where MKI suppresses the induction of transcription by DNA damage, estrogen signaling, NF-κB activation, and several other transcription-activating signals (7–11).

For most of the affected genes and signal transduction pathways, MKI enhanced the effects of castration, indicating that Mediator kinase activity restrains castration-induced transcriptional reprogramming, as has been previously shown for its effect on chemically induced cell fate reprogramming (12). In some cases, the genes or pathways were downregulated by a combination of castration and MKI. This category included MYC target genes, some (but not all) of which positively regulate cell proliferation. In contrast, MYC itself was downregulated by castration but not affected by MKI, indicating that CDK8/19 exerted its effects downstream of MYC. The MYC pathway, a major driver of PCa (64), was reported to be positively regulated by Mediator kinases in other cell types (65, 66). To determine whether MKI inhibits MYC-driven CRPC, we have tested the effects of CDK8/19i treatment on in vivo growth of MYC-CaP-CR (58), a castration-resistant derivative of the MYC-driven transgenic PCa model MYC-CaP (59). CDK8/19i treatment almost completely suppressed the growth of MYC-CaP-CR tumors, with or without castration, identifying the MYC pathway as one of the transcriptional targets for CRPC suppression by MKI.

The largest group of castration- and MKI-affected genes, as well as transcription factor pathways, were induced by castration and further upregulated by MKI. The effect of MKI on castration-induced gene expression closely resembles the hyperinduction of super-enhancer–associated genes by MKI in AML cells, where the resulting unbalanced expression of such genes inhibits cell proliferation (13). Remarkably, ETV6 and FOSL2, 2 of 6 MKI-hyperinduced genes in AML cells, overexpression of which was shown to inhibit AML cell growth (13), were also upregulated by MKI in 22Rv1 tumors. It remains to be determined whether the genes induced by castration and MKI in CRPC tumors in vivo are also associated with super-enhancers, as in the case of AML. This seems likely, since super-enhancers found in PCa are enriched in the Mediator complex (67), and the levels of this complex are increased by MKI (8). Tumor suppression by either downregulation or upregulation of key transcriptional signals is well established in PCa, where both androgen deprivation and supraphysiological doses of testosterone (SupraT) suppress the growth of cancers with canonical AR signaling, the basis of bipolar androgen therapy (68). While CRPC with altered AR signaling (such as 22Rv1) is not sensitive to SupraT, hyperinduction of castration-responsive genes by MKI resembles SupraT in producing a strong tumor-suppressive effect.

In addition to the transcriptomic effects on tumor cells, we found that MKI affected stromal gene expression in 22Rv1 CRPC in a way that could contribute to tumor suppression. Several stromal genes upregulated by MKI were shown to have tumor-suppressive effects in the stroma (Igfbp4 [ref. 53], Ccdc80 [ref. 54], Dlk1 [ref. 55]), and some downregulated genes were reported to have tumor-supporting activities (Ramp3 [ref. 56], Osm [ref. 57]). Remarkably, many of the stromal genes were affected not only by systemic treatment with a CDK8/19i but also by Mediator kinase mutagenesis in tumor cells alone, suggesting that CDK8/19 activity in the tumor cells molds the tumor microenvironment.

The role of the tumor environment in CRPC response to MKI was also suggested by 2 long-term (300-day) studies, in which 22Rv1 xenografts in castrated nude mice were treated with SNX631 either for the first 38 days or for the entire 300-day period (such an exceptional length of continuous drug treatment reflects the lack of toxicity of selective CDK8/19i). Long-term treatment or long-term follow-up after a shorter treatment period revealed not only tumor growth inhibition but also regression, with 17%–25% of tumors disappearing and not recurring until the end of the 300-day period, indicating the achievement of cures. To the best of our knowledge, there are no other reports of cures achieved in 22Rv1 or other CRPC models.

Interestingly, tumor regression and cures were observed in NCr nude mice that lack mature T cells but still have B cells and robust NK cell responses. On the other hand, we did not observe tumor regression in NSG mice, which are B, T, and NK cell deficient with impaired innate immunity. Antitumor activity of NK cells (20, 21), as well as effector T cells (22), is known to be stimulated by MKI. To test the role of immune stimulation by MKI in CRPC suppression, we compared the effects of the same CDK8/19i on murine MYC-CaP-CR CRPC tumors growing in immunocompetent FVB and immunodeficient NSG hosts. Tumor suppression of MYC-CaP-CR appears to be more complete in the immunocompetent mice, suggesting that immune stimulation could have contributed to tumor suppression in this model.

CDK8/19i treatment also suppressed tumor growth in 3 AR-positive PDX models derived from PCa patients, at least 2 of whom failed ADT and chemotherapy. Interestingly, the effect of CDK8/19 inhibition in one of these models was associated with the suppression of intratumoral blood supply, indicating a stromal effect of MKI. In a related example, the positive regulation of angiogenesis by CDK8 was previously suggested in pancreatic cancer (69). Interestingly, PDX suppression by CDK8/19i was observed in intact mice, to a greater degree than in the 22Rv1 model, as also seen in MYC-CaP-CR and C4-2 xenografts. Hence, the requirement of castration for the effect of CDK8/19i on CRPC is not absolute but depends on the specific tumor. From a clinical standpoint, however, the responses observed under the conditions of androgen deprivation should be especially relevant, since most CRPC patients would have undergone chemical castration.

Remarkably, the gene signature reflecting Mediator kinase activity in 22Rv1 tumors showed a correlation with the progression from primary PCa to mCRPC, as well as a strong correlation with shorter OS in mCRPC patients. These correlations suggest that pharmacological inhibition of CDK8/19 should have a survival benefit in mCRPC. Taken together, our results support the development of Mediator kinase inhibitors as a new class of drugs for the treatment of CRPC that is resistant to currently available therapies.

## Methods

The sources of all the cell lines, reagents, and software are listed in Supplemental Table 5, and all the procedures are described in detail in Supplemental Methods. Primers for reverse transcription qPCR analysis are shown in Supplemental Table 6. All raw and processed RNA-Seq data were uploaded to GEO (see *Data availability*). Individual RNA-Seq sample information is listed in Supplemental Table 7.

### Sex as a biological variable.

Our study exclusively examined male mice because the disease modeled (prostate cancer) is only relevant in males.

### Statistics.

RNA-Seq experiments were conducted with a minimum of 3 biological replicates for each treatment condition. The slope and Pearson’s correlation coefficients were determined through linear regression and correlation analysis using GraphPad Prism 9 software. qPCR analysis was performed in biological triplicates, and the data are presented as the mean ± SEM. Statistical significance was evaluated by ordinary 2-way ANOVA and Tukey’s multiple-comparison test using GraphPad Prism 9 software. A 2-tailed Student’s *t* test was performed for comparisons between only 2 groups. The log-rank (Mantel-Cox) test, implemented in GraphPad Prism 9 software, was used to assess statistical significance between survival curves in the Kaplan-Meier analysis. A *P* value of less than 0.05 was used to determine statistical significance.

### Study approval.

All experimental animal procedures were performed according to guidelines approved by the Institutional Animal Care and Use Committees (IACUCs) at the study sites indicated in Supplemental Methods and were conducted in accordance with the NIH *Guide for the Care and Use of Laboratory Animals* (National Academies Press, 2011).

### Data availability.

Requests for further information should be directed to corresponding author MC. The RNA-Seq data were deposited in the Gene Expression Omnibus database (GEO GSE240167, GSE240369, and GSE240370) and are publicly available. Any additional information required for reanalyzing the data is available from MC upon request.

## Author contributions

MC and IBR conceived the project. MC, IBR, MBL, CM, LE, XZ, DCP, and EVB oversaw the project, designed the experiments, and interpreted the data. MC, IBR, and J Li wrote the manuscript. GW, EVB, and BG reviewed and edited the manuscript. J Li, TAH, MC, YL, LW, J Liang, VS, HJ, LZ, CC, XD, ZTM, KRK, CED, GPS, CL, and AAC performed experiments. MC, J Li, HJ, and BG carried out bioinformatics analysis.

## Supplementary Material

Supplemental data

Unedited blot and gel images

Supplemental tables 1, 2, 3, 4, 6 and 7

Supporting data values

## Version 1 03/28/2024

In-Press Preview

## Version 2 05/15/2024

Electronic publication
