# Supplementary material for: Mediator kinase inhibition reverses castration resistance of advanced prostate cancer
Source: J Clin Invest. 2024 Mar 28;134(10):e176709. doi: 10.1172/JCI176709 (PMC11093614; doi:10.1172/JCI176709)
Supplement: Supplemental data [file jci-134-176709-s023.pdf]

Supplementary Materials for

Mediator kinase inhibition reverses castration resistance of advanced prostate cancer

Jing Li[1], Thomas A. Hilimire[1,2], Yueying Liu[3], Lili Wang[1], Jiaxin Liang[1], Balazs Gyorffy[4,5], Vitali Sikirzhytski[1], Hao Ji[1], Li Zhang[1], Chen Cheng[1], Xiaokai Ding[1], Kendall R. Kerr[1], Charles E. Dowling[1], Alexander A. Chumanevich[1], Zachary T Mack[1], Gary P. Schools[1], Chang-uk Lim[1], Leigh Ellis[6,7], Xiaolin Zi[6], Donald C. Porter[2], Eugenia V. Broude[1], Campbell McInnes[1], George Wilding[2], Michael B. Lilly[3], Igor B. Roninson[1]*, Mengqian Chen*[1,2]

Correspondence to: Igor Roninson roninsoni@cop.sc.edu; Mengqian Chen chenm@cop.sc.edu,

This PDF file includes:

Supplementary Materials and Methods

Figs. S1 to S9

Table S5

Other Supplementary Materials for this manuscript include the following:

Tables S1, S2, S3, S4, S6 and S7 (Each Table is a tab in an Excel spreadsheet)

Supporting data values for all graphs (except for bioinformatic analysis)

Unedited blot/gel images

Supplementary Materials and Methods

Cell lines

HEK293 (293), a derivative of 293 with CDK8/19 double-knockout (293-dKO), VCaP, LNCaP, LNCaP-C4-2, LNCaP-LN3, DU145, PC-3, MYC-CaP-CR, 22Rv1 (Rv1-WT) and 22Rv1 derivatives were acquired from sources listed in Table S5. LNCaP and its derivatives C4-2 and LN3, as well as 22Rv1 and its derivatives, were cultured in RPMI-1640 medium supplemented with 2 mM L-glutamine, 1 mM sodium pyruvate, 1.5 g/L sodium bicarbonate, 10 mM HEPES, 0.45% D-glucose, 10% fetal bovine serum (FBS) or charcoal-stripped FBS (CSS), and 1% penicillin-streptomycin (P/S). This combination is referred to as PCa FBS medium when using FBS and PCa CSS medium when using CSS. HEK293 and its derivatives, VCaP and MYC-CaP-CR were cultured in DMEM medium supplemented with 10% FBS and 1% P/S. DU145 cells were cultured in EMEM medium supplemented with 10% FBS and 1% P/S. PC-3 cells were cultured in F-12K medium supplemented with 10% FBS and 1% P/S. All the cells were cultured at 37°C with 5% CO_2. Regular testing for mycoplasma contamination was conducted every 3-4 weeks using a MycoAlert PLUS Mycoplasma Detection Kit (Lonza). All cell lines and derivatives were authenticated by STR matching assays performed by the functional genomics core (FGC) of the Center for Targeted Therapeutics (CTT) at the University of South Carolina (USC).

Generation of 22Rv1 derivatives

22Rv1 (Rv1-WT) cells were transduced with pHIV-Luc-BlastR to generate the derivative Rv1-Luc. Rv1-dKO cells, previously generated using lentiCRISPR-Puro-sgCDK8 and lentiCRISPR-Blast-sgCDK19 constructs (Table S5), were transduced with a set of pHIV-dTomato-based constructs, including control, CDK19_CasR, and CDK19M_CasR variants (Table S5). Following two rounds of fluorescence-based sorting with the FACS Aria III (BD Biosciences) at the Microscopy and Flow Cytometry Core (MFCC) of CTT at USC, Rv1-dKO-V, Rv1-dKO-19, and Rv1-dKO-19M cells were obtained. Rv1-dKO-V cells were then transduced with pHIV-ZsGreen-CDK8_CasR and pHIV-ZsGreen-CDK8M_CasR constructs, sorted using flow cytometry, and subcloned. The clones with validated CDK8 or CDK8M expression were pooled together and designated as Rv1-dKO-8 and Rv1-dKO-8M. Rv1-dKO-19 and Rv1-dKO-19M cells were transduced with the same CDK8/8M_CasR constructs and sorted to establish the Rv1-dKO-19-8, Rv1-dKO-19-8M, Rv1-dKO-19M-8 and Rv1-dKO-19M-8M populations.

The lentiviral constructs pHIV-dTomato-CDK19_CasR and pHIV-dTomato-CDK19M_CasR, which contained the coding sequences of human CDK19-wild-type or CDK19-D173A kinase-inactive mutant with mutations introduced in the sgRNA-targeting sequence and proximal PAM sequences (blocking sgRNA targeting but not changing the encoded protein sequences), were generated using the Phusion Site-Directed Mutagenesis Kit (Thermo Scientific) with the hCDK19-CasR-F/R primers (listed in Table S6) and the original pHIV-dTomato-CDK19/CDK19M constructs as cloning templates. To obtain the CDK8/8M_CasR constructs, the pHIV-ZsGreen-based vectors (pHIV-ZsGreen-CDK8 and pHIV-ZsGreen-CDK8M) were first generated by subcloning the coding sequences of human CDK8-wild-type or CDK8-D173A kinase-inactive mutants from the pHIV-dTomato-CDK8/CDK8M constructs and then used as templates for mutagenesis with hCDK8-CasR-F/R primers (Table S6) to achieve the Cas9-resistance. All lentiviruses were produced by the FGC.

In vitro cell growth assays

LNCaP-C4-2 and 22Rv1 derivatives were plated on P100 plates in FBS or CSS media. After overnight attachment, the cells were treated with vehicle control (0.1% DMSO) or the test compounds at the indicated concentrations. For long-duration experiments, the culture media, including the drugs, were refreshed every 3 days. At the end of the incubation period, the cells were washed with 1X PBS, trypsinized, and harvested. Cell counts were determined using the TC20 Automated Cell Counter (Bio-Rad) on Dual-Chamber Cell Counting Slides (Bio-Rad) and Trypan Blue Dye (Bio-Rad). The effects of CDK8/19 inhibitors at different concentrations on the growth of LNCaP and Rv1 derivatives in FBS or CSS media were also measured by Sulforhodamine B (SRB) assay in 96-well plates, following different treatment durations.

PSA ELISA analysis

For PSA ELISA analysis in vitro, cells were seeded into 12-well plates and incubated overnight to allow adhesion and recovery. The cells were then exposed to different concentrations of CDK8/19 inhibitors (CDK8/19i) for 72 h before the culture medium was harvested for analysis. For the PSA ELISA in vivo assays, mice bearing xenograft tumors were treated with either a vehicle control or CDK8/19i, and sera were collected post-treatment. PSA levels were quantified in both conditioned media and mouse sera using the PSA ELISA kit (Abcam), according to the manufacturer's instructions.

Western blot analysis

Control and treated cells grown in P100 plates were washed twice with cold PBS and then lysed in RIPA lysis buffer (50 mM Tris-HCl, pH 8.0; 150 mM NaCl; 5 mM EDTA; 0.5 mM EGTA; 1% Igepal CA-630 (NP-40), 0.1% SDS, and 0.5% sodium deoxycholate) or IP lysis buffer (50mM HEPES, pH 7.5; 140mM NaCl, 1% TX-100, 0.5% sodium deoxycholate and 1mM EDTA) supplemented with 1x protease/phosphatase inhibitor cocktail (Thermo Scientific #78438), 2 mM Na_3VO_4, and 10 mM NaF. Ice-cold lysates were briefly sonicated (5-10 seconds each time) 3 times to solubilize chromatin proteins, followed by centrifugation (14,000 × g, 10 min). The protein concentration was determined using a DC protein assay (Bio-Rad). Lysate samples with the same amount of total protein (40-50 µg) were mixed with 4x Laemmli Sample Buffer (Bio-Rad #161-0737, supplemented with 2-mercaptoethanol) and electrophoresed on 4-12% Express-Plus PAGE gels in Tris-MOPS (SDS) running buffer (GenScript #M00138). Proteins were transferred onto polyvinylidene fluoride (PVDF) membranes, which were then blocked with 5% non-fat milk and incubated with primary and secondary antibodies (listed in Table S5). Protein bands were visualized using the Western Lighting Plus ECL detection reagent (Perkin Elmer, Waltham, MA, USA) and imaged using the ChemiDoc Touch™ system (Bio-Rad). Image processing and densitometry analyses were conducted using ImageLab software (Bio-Rad).

Mouse xenograft studies

All mice were maintained under pathogen-free conditions, and random assignment was employed to distribute the animals across the different experimental groups in each study. Most studies were performed in male NSG (NOD.Cg-Prkdcscid/Il2rgtm1Wjl/SzJ, The Jackson laboratory) or NCr nude (CrTac:NCr-Foxn1nu, Taconic) or FVB (FVB/NTac, Taconic) mice at the University of South Carolina (USC). The efficacy study in the PDX-SM0310 and PDX-CG0509 models was performed in male NSG mice at the

Medical University of South Carolina. The efficacy study in the C4-2 xenograft model was performed in male NCG mice (NOD-Prkdcem26Cd52 /Il2rgem26Cd22 /NjuCrl, Nanjing Galaxy Biopharmaceutical Co., Ltd) by Crown Bioscience (Beijing) Inc. Detailed experimental procedures of each in vivo animal study are described below.

The efficacy study in the C4-2 xenograft model was performed in male NCG mice (NOD-Prkdc^{em26Cd52} /Il2rg^{em26Cd22} /NjuCrl, Nanjing Galaxy Biopharmaceutical Co., Ltd) by Crown Bioscience (Beijing) Inc. under the IACUC-approved protocol. C4-2 cells (5x10^6 cells in 0.1 mL 50% Matrigel per animal) were inoculated s.c. at the right front flank into intact male NCG mice (8-9 weeks old, 22-31 grams). On day 10, 20 animals were randomly allocated to two study groups. The mean tumor size at randomization was approximately 130 mm^3. Randomization was performed based on "Matched distribution" randomization method (Study Director TM software, version 3.1.399.19). The animals in the control group were dosed with vehicle (0.05% carboxymethyl cellulose solution, 10 mL/kg, p.o., q.d.) and the animals in the treatment group were dosed with SNX631 (2.5 mg/mL suspension formulation in the vehicle, 10 mL/kg, p.o., b.i.d.) at 50 mg/kg/day. The animals were continuously treated for 11 days, and the xenograft tumors were excised and weighed at the end of the study. Endpoint serum samples were collected for PSA ELISA.

Studies with 22Rv1 cells were performed at the University of South Carolina (USC) Department of Laboratory Animal Research (DLAR), under IACUC-approved procedures. For tumor growth analysis of 22Rv1 derivatives, cells were inoculated s.c. (2x10^6 cells in 0.1 mL 50% Matrigel per animal) in the right flank of intact or castrated male NSG mice (9-11 weeks old). Castration was performed by either surgical orchiectomy (for Rv1-WT and Rv1-dKO studies) or degarelix treatment (administered s.c. at 10 mg/kg monthly for Rv1-Luc, dKO-V, dKO-8, dKO-8M, dKO-19, and dKO-19M studies), and tumor inoculation was done 10-14 days after castration. Tumor dimensions and body weights of the mice were recorded twice per week. Upon reaching the humane endpoint, tumors were collected and stored for further studies, including RNA-Seq (stabilized in RNA-later stabilization solution), IHC (fixed in 10% neutralized formalin for 24-48 hours, then stored in 75% ethanol), and protein analysis (snap-frozen and stored at -80°C).

For Degarelix treatment, Rv1-Luc and 22Rv1-dKO derivative cells were inoculated s.c. (2x10^6 cells in 0.1 mL 50% Matrigel per animal) in the right flank of male NSG mice (9-11 weeks old). Once the tumor volumes reached ~150mm^3, the animals were randomly assigned to the control or treatment groups and treated with PBS or Degarelix (administered s.c., 10 mg/kg once a month). Tumor sizes and body weights were measured 2-3 times per week until the tumors reached the humane endpoints. The effects of degarelix treatment were evaluated using Kaplan-Meier analysis, wherein a tumor size > 1500 mm^3 was considered as a survival endpoint.

For SNX631 treatment of 22Rv1 xenografts, Rv1-WT and Rv1-Luc cells were inoculated s.c. (2x10^6 cells in 0.1 mL 50% Matrigel per animal) in the right flank of intact or castrated male NSG mice (8-10 weeks old). Castration was performed by either surgical orchiectomy (for the Rv1-WT study) or Degarelix treatment (administered s.c. at 10 mg/kg monthly, for the Rv1-Luc study), and tumor inoculation was done 10-14 days post castration. Animals were randomly allocated to two study groups when the mean tumor size reached 100-200 mm^3. For the Rv1-WT study, the treatment group received an SNX631-medicated diet (500 ppm, producing 30-60 mg/kg/day dosage on average) and the control group received the control diet. For the Rv1-Luc study, the animals in the control group were treated with vehicle (70% PEG-400/30%

Propylene Glycol, 5 mL/kg, p.o., b.i.d.) and the treatment group was administered SNX631 (6 mg/mL solution in the vehicle, 5 mL/kg, p.o., b.i.d.) at 60 mg/kg/day. Tumor dimensions and mouse body weights were measured 2-3 times per week. At the endpoints, tumors were collected and stored for further studies, including RNA-Seq (stabilized in RNA-later stabilization solution), IHC (fixed in 10% neutralized formalin for 24-48 hours, then stored in 75% ethanol), and protein analysis (snap-frozen and stored at -80°C).

For long-term SNX631 treatment, Rv1-WT cells were inoculated s.c. (2x10^{6} cells in 0.1 mL 50% Matrigel per animal) in the right flank of castrated male NCr nude mice (8-10 weeks old). Animals were randomly allocated to four study groups (one control group and three treatment groups at different dosages) when the mean tumor size reached 100-200 mm^{3}. In the first long-term study, the control group animals were dosed with vehicle (0.05% carboxymethyl cellulose solution, 10 mL/kg, p.o., q.d.), and the animals in the treatment groups were administered SNX631 (in the suspension vehicle at 2.5 or 5 mg/mL, 10 mL/kg, p.o., q.d., or b.i.d.) at 50 or 100 mg/kg/day. The animals were treated for 38 days and left untreated for the remainder of the study period. In the second long-term study, the control group animals were dosed with vehicle (70% PEG-400 / 30% Propylene Glycol, 5 mL/kg, p.o., b.i.d.) and the animals in the treatment groups were administered SNX631 (in the vehicle solution at 3 or 6 mg/mL, 5 mL/kg, p.o., q.d., or b.i.d.) at 30 or 60 mg/kg/day. After 29 days of treatment, the control group animals were switched to the q.d. dosing regimen and animals of all the treatment groups were switched to a 30 mg/kg/day dosing regimen for another 34 days and then were placed on either control or SNX631-mediated chow (350 ppm), respectively, for the remaining period of the study. Tumor sizes and body weights were measured 2-3 times per week until the tumors reached the humane endpoints. The effects of long-term SNX631 treatment were assessed using Kaplan-Meier analysis.

For SNX631-6 treatment of MYC-CaP-CR syngeneic allograft tumor models, MYC-CaP-CR cells were inoculated s.c. (0.5x10^{6} cells in 0.1 mL 50% Matrigel per animal) in the right flank of intact or castrated male FVB mice or intact male NSG mice (8-10 weeks old). Castration was performed by Degarelix treatment (administered s.c. at 10 mg/kg monthly), and tumor inoculation was done 10-14 days post castration. Animals were randomly allocated to two study groups when tumors are palpable. The treatment group received an SNX631-6-medicated diet (500 ppm, producing 30-60 mg/kg/day dosage on average) and the control group received the control diet. Tumor dimensions and mouse body weights were measured 2-3 times per week. At the endpoints, tumors were excised and weighed at the end of the study.

Studies with PDX-SM0310 and PDX-CG0509 models were performed at the Medical University of South Carolina (MUSC), under IACUC-approved protocol. These PDX models were established at the University of California, Irvine. PDX tissues were dissociated into single-cell suspensions by combining mechanical dissociation with enzymatic degradation of the extracellular matrix. The tumor tissues were enzymatically digested using the Tumor Dissociation Kit (Miltenyi Biotec), and a GentleMACS™ Octo Dissociator with Heater was used for the mechanical dissociation steps. After dissociation, the sample was passed through a 70 µm strainer and washed with 20 mL RPMI 1640 medium. The resulting single-cell suspension was inoculated s.c. (1x10^{6} cells in 0.1 mL 50% Matrigel per animal) in the right flank of male NSG mice. For PDX-SM0310 studies, animals were chemically castrated with degarelix (administered s.c. at 10 mg/kg)

two weeks after tumor inoculation. Animals were randomly allocated to two study groups when the mean tumor size reached 50-100 mm^3. The treatment group received an SNX631-medicated diet (500 ppm) and the control group received a control diet. Tumor sizes and body weights were measured 2-3 times per week until the tumors reached the humane endpoints. The effects of long-term SNX631 treatment were assessed using Kaplan-Meier analysis.

Studies with PDX-J000077451 model (from Jackson Laboratory) were performed at USC, under IACUC-approved protocol. Male NSG mice (8-10 weeks old) were inoculated by taking a 10 mm^3 tumor piece excised from freshly collected xenograft tissue and implanted subcutaneously in the right flank. Once the tumors reached 150 mm^3, mice were randomized into two study groups. The treatment group received SNX631-6 in a medicated diet (500 ppm, 30-60 mg/kg/day dosage on average) and the control group received a control diet. Tumor dimensions and mouse body weights were measured twice per week. On the final day of the study, the tumors were excised, weighed, imaged, and stored for further studies.

RNA analysis

RNA from cell culture was extracted using the RNeasy Mini Kit (Qiagen) and RNA from tumor tissues was extracted using the mRNeasy Mini Kit (QIAGEN), according to the manufacturer's protocols. RT-qPCR analysis was conducted with primers listed in Table S6 using cDNA synthesis reagents and SYBR Green Supermix qPCR reagents listed in Table S5. RNA-Seq analysis, library preparation, next-generation sequencing (NGS), and post-processing of raw data were performed by the FGC. Processed reads were then aligned to a hybrid reference genome consisting of both the human GRCh38.p13 primary assembly genome and mouse GRCm39 primary assembly genome. Differential expression (DE) analysis was performed in R, using the DESeq2 pipeline. All raw and processed RNA-Seq data were uploaded to GEO (see Data availability). Individual RNA-Seq sample information is listed in Table S7.

LNCaP cells were seeded in 12-well plates at a density of 3 X 10^5/well and cultured in PCa CSS medium for 72 h. In study 1, cells were treated (in triplicate) with different concentrations of R1881 (0.1, 1, 10 nM) for 24 h. In study 2, cells were treated with DMSO (0.1%), Senexin B (1 µM), R1881 (0.1 nM), or a combination of Senexin B and R1881 for 24 h. In study 3, cells were treated with DMSO (0.1%), SNX631 (500 nM), R1881 (1 nM), or a combination of SNX631 and R1881 for 72 h. The effects of CDK8/19 inhibition on gene expression in 22Rv1 derivatives were evaluated as follows: (1) under androgen-supplemented conditions, cells were seeded into 12-well plates at a density of 2 X 10^5/well in PCa FBS medium, incubated overnight, and then treated with 0.1% DMSO (vehicle control) or 500 nM SNX631 in FBS medium for 3 days. (2) Under androgen-deprived conditions, cells were first subcultured in PCa CSS media for one passage (3-4 days) and then seeded into 12-well plates at a density of $3x10^5$ cells/well in PCa CSS media, incubated overnight, and then treated with 0.1% DMSO (vehicle control) or 500 nM SNX631 in CSS media for 3 days. RNA was extracted from the cell culture using the RNeasy Mini Kit (Qiagen), according to the manufacturer's protocol.

For tumor tissue RNA extraction, tumor xenografts were dissected from euthanized animals using sterilized surgical tools. Pieces of non-necrotic tumor tissue ~100mm^3 were excised at the margin near the midline section and submerged in 500 µL RNA-Later stabilization solution (Thermo Fisher Scientific) at room temperature. After stabilization, RNA samples were extracted from the tumor tissues using the mRNeasy Mini Kit (QIAGEN) according to the manufacturer's protocol.

For RT-qPCR analysis, RNA samples (500 ng each) were used for cDNA synthesis using an iScript cDNA Synthesis Kit (Bio-Rad). Subsequently, the expression levels of target genes were quantified using iTaq Universal SYBR Green Supermix and the CFX384 Real-Time PCR Detection System (Bio-Rad). The primers used for qPCR are listed in Table S6. Data derived from real-time PCR were processed using Bio-Rad CFX Manager software to extract Ct values from the qPCR reactions. The relative expression levels of specific genes were computed using the following formula: Relative Expression= $2^{(Ct\_{Reference} - Ct\_{Target}\ gene)}$, where RPL13A served as the reference gene.

For RNA-seq analysis, library preparation, next-generation sequencing (NGS), and post-processing of raw data were performed by the Functional Genomics Core (FGC) of the Center for Targeted Therapeutics (CTT). RNA-Seq libraries were generated using the NEBNext Ultra II Directional RNA Library Prep Kit and sequenced on HiSeq 3000/4000 (Genewiz, Inc., South Plainfield, NJ) or Illumina NovaSeq (MedGenome, Inc., Foster City, CA) platforms, utilizing paired-end sequencing. Demultiplexed raw reads data were initially processed using Cutadapt software (v 2.8) to trim off the adaptor sequences and filter out reads that possessed short insert sequences. Processed reads were then aligned to a hybrid reference genome consisting of both the human GRCh38.p13 primary assembly genome and mouse GRCm39 primary assembly genome using STAR (v2.7.2). The alignment output BAM files were further processed using the featureCounts software from the Subreads package, with the hybrid annotation file built from gencode.v41.annotation.gtf and gencode.vM30.annotation.gtf annotation files downloaded from the GENCODE website to generate the count matrix data. Differential expression (DE) analysis was performed in R, using the DESeq2 package. Within the DESeq2 pipeline, the normalized count data were fitted to a negative binomial distribution model using a generalized linear model (GLM) framework, and the Benjamini-Hochberg procedure was used to control the false discovery rate (FDR) for multiple testing. The log2 fold change (logFC) was calculated by computing the base-2 logarithm of the ratio of the mean normalized expression value (transcripts per million, TPM) for cell culture samples or the median normalized expression value (TPM) for xenograft tissues for each group. A small pseudocount was added to the expression values of all genes before calculating the logFC to avoid division by zero. The logFC and FDR values calculated from the DESeq2 pipeline were used to select differentially expressed genes (DEGs). To select high-confidence DEGs from multiple RNA-Seq experiments, the following criteria were applied: (1) FDR < 0.01 in all experiments; (2) average log2FC from multiple experiments > log2(1.5). Gene set enrichment analysis (GSEA) for different comparisons was conducted using the fgsea package with specific gene sets downloaded from the Human Molecular Signatures Database (MSigDB). All raw and processed RNA-Seq data were uploaded to GEO (see Data Availability section). Detailed information about the individual RNA-Seq samples (sample title and description, GEO accession number) is listed in Table S7.

Bioinformatic analysis of clinical samples

For the tumor-normal comparison, RNA-Seq data from TCGA and Genotype-Tissue Expression (GTEx) portals were used. Data processing and analysis features were executed in the R statistical environment. Read counts were DESeq2 normalized, followed by a second scaling normalization. The biomaRt and AnnotationDbi R packages were used to select and annotate the appropriate genes in each dataset. A comparison between the normal and tumor samples was performed using Mann–Whitney U test. The statistical significance cut-off was set at $p < 0.01$.

RNA-Seq data from clinical prostate cancer patient samples were retrieved from TCGA database (TCGA-PRAD project) and cBioportal database (SU2C prostate cancer project) using the R packages TCGAbiolinks and cBioPortalData. A total of 52 normal prostate tissues, 501 primary prostate cancer tissues, and 266 mCRPC tissues were included in the analysis. Log2-transformed FPKM values plus pseudo-number 1 (log2(FPKM + 1)) were used for expression analysis. The correlation between genes was analyzed using the ggcor package.

To assess the correlation between Mediator kinase activity and gene expression changes during PCa carcinogenesis and progression, we generated a signature set of 266 genes (Table S4) that were affected by MKI in 22Rv1 CRPC xenograft tumors, selected from the 315 DEGs listed in Table S3 and expressed (fpkm >0.1) in the RNA-Seq data of clinical samples from primary PCa patients (n= 497) and mCRPC patients (n=81) in TCGA-PRAD and SU2C projects, where overall survival data were available. Correlation analysis was performed to compare the fold changes in the expression of this set of genes in 22Rv1 tumors in the absence vs the presence of MKI and the changes in their expression across clinical conditions. To assess the correlation between Mediator kinase activity and survival of PCa patients, the expression of 266 genes was first transformed into z-scores, and the signature score for each patient was calculated by subtracting the cumulative z-scores of the genes upregulated by MKI from the cumulative z-scores of the genes downregulated by MKI. The correlation of the signature score with overall survival among primary PCa and mCRPC patients was assessed by Kaplan-Meier analysis using the Survminer package. To ensure accuracy, the cut-off values for the Kaplan-Meier plots were selected based on the lowest p-value within the interquartile range (25th to 75th percentile) of the gene signature scores among the patient cohort.

Histology and Image analysis

For machine learning-based H&E analysis, tumor samples harvested from the mice were washed with PBS and fixed in 10% neutralized paraformaldehyde for 24-38 hours. The samples were washed twice with PBS and stored in 70% ethanol at 4°C. Fixed tumors were embedded in paraffin and sectioned into 5 µm sections. H&E staining was performed according to the standard procedures. The H&E-stained tumor sections were imaged using a Leica DMIRE fluorescence microscope, controlled via an Inscoper control block with the Inscoper 6.3.0 software package. The images were collected using a 10x Phase objective and Zeiss AxioCam MRC color camera. The acquisition of single images (tiles) was conducted using a Marzhauser Wetzlar SCAN IM 120 × 100 XY stage. Single images were stitched and corrected for brightness and contrast levels using custom ImageJ scripts. The machine learning platform WEKA v.3.3.2 was utilized to differentiate the proliferation, apoptosis, and necrosis areas based on the characteristic morphologies. Artificial Intelligence (AI) models were created using manually selected reference sets of images representing proliferation, apoptosis, and necrosis using rounds of reference dataset optimization, training of AI models, and accurate validation of AI-based automatic image segmentation using test images not included in the training subsets. Finally, custom ImageJ scripts were used to apply the developed AI model to the whole tissue sections. The results of the AI-based detection of characteristic phenotypes within all tissue sections were confirmed by experienced pathologists. For visualization purposes, areas assigned to proliferation, apoptosis, and necrosis were marked with green, yellow, and red, respectively. Quantification of the detected areas was performed using WEKA-generated probability maps and a customized ImageJ threshold tool. All preliminary calculations were done using Precision 5820 station: Intel® Core™ i9, 18 cores, 36 threads, 3.00 GHz to 4.80 GHz Turbo, AMD® Radeon™ Pro WX 3200,

4 GB GDDR5, 4 mDP, 128 GB DDR4 RAM, and M2 PCIe NVMe Class 40 SSD. computationally heavy steps were performed using a 1060 TeraFLOPS computer cluster (Research Computing, USC) with a current capacity of 1.4 petabytes.

For immunofluorescence analysis, immunostaining was performed on sections cut from formalin-fixed, paraffin-embedded tissue blocks. The sections were deparaffinized and hydrated using the following steps:10 min in xylene (twice), 7 min in 100% ethanol (twice), 7 min in 70% ethanol, and 5 min in water at room temperature (three times). Antigen retrieval was performed by boiling the samples in water for 20 min. Deparaffinized and hydrated tumor sections were autoclaved at 121°C for 20 min in 10 mM sodium citrate (pH 6), blocked with 5% normal donkey serum in PBS plus Tween 20 (PBST), then incubated overnight at 4°C in a 1 to 1000 dilution of rabbit anti-AR (Santa Cruz). Sections were then washed in PBST and incubated with a 1:200 dilution of Alexa Fluor 555 donkey anti-rabbit IgG (Thermo Fisher) for 1 h. Afterwards, the sections were washed in PBST, counterstained for 20 min with 1 µM DAPI, and mounted under coverslips with Prolong Glass (Thermo Fisher). Representative tumor areas were imaged with a 20x objective using the Agilent Cytation 5 imaging system. Tiff files were exported from Agilent Gen5.12 software, and ImageJ was used to create maximum intensity projections (MIPs).

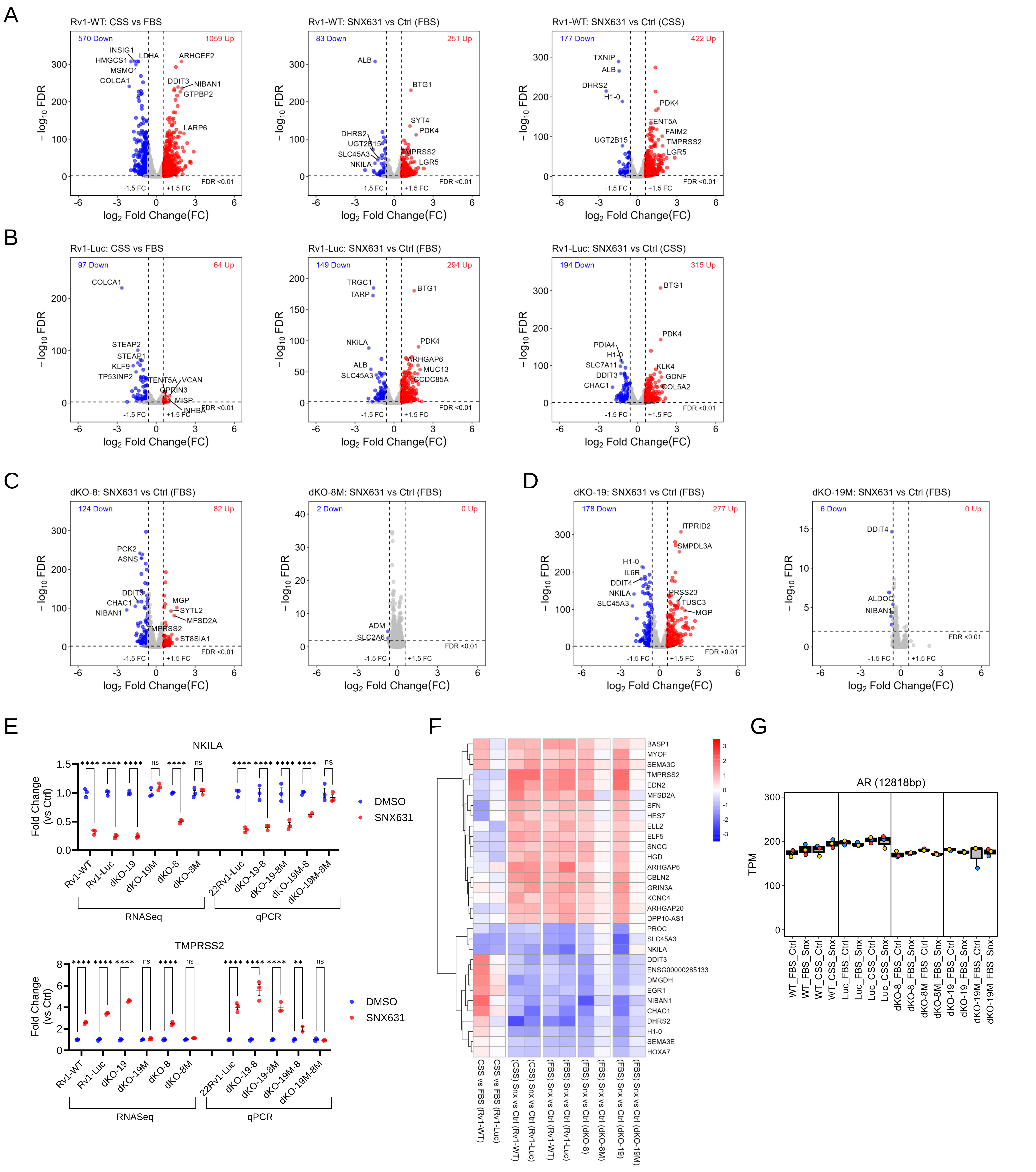

Figure S1. RNA-Seq analysis of the effects of Mediator kinase inhibition (MKI) on gene expression in 22Rv1 derivatives *in vitro*.

(A-B) Effects of MKI (SNX631 vs Ctrl) androgen-containing (FBS) and androgen-deprived (CSS) media and the effects of androgen deprivation (CSS vs FBS) in the absence or presence of SNX631 in Rv1-WT **(A)** and Rv1-Luc **(B)** cells. **(C-D)** Effects of MKI (SNX631 vs Ctrl) on dKO-8 and dKO-8M **(C)** and dKO-19 and dKO-19M **(D)** derivatives in FBS media. DEGs passing the selection criteria (FC >1.5, FDR <0.01) are marked with red (upregulated) and blue (downregulated) dots. **(E)** Effects of MKI on RNA expression of CDK8/19-dependent genes, determined by RNA-Seq or QPCR analysis, in 22Rv1 derivatives. **(F)** Heatmap of 33 DEGs regulated by SNX631 in common in Rv1-WT, Rv1-Luc, dKO-8 and dKO-19 cells. **(G)** AR RNA expression under different conditions. Asterisks mark p-values: 0 < ****< 0.0001 < ***< 0.001 < **< 0.01< *< 0.05. ns, not significant.

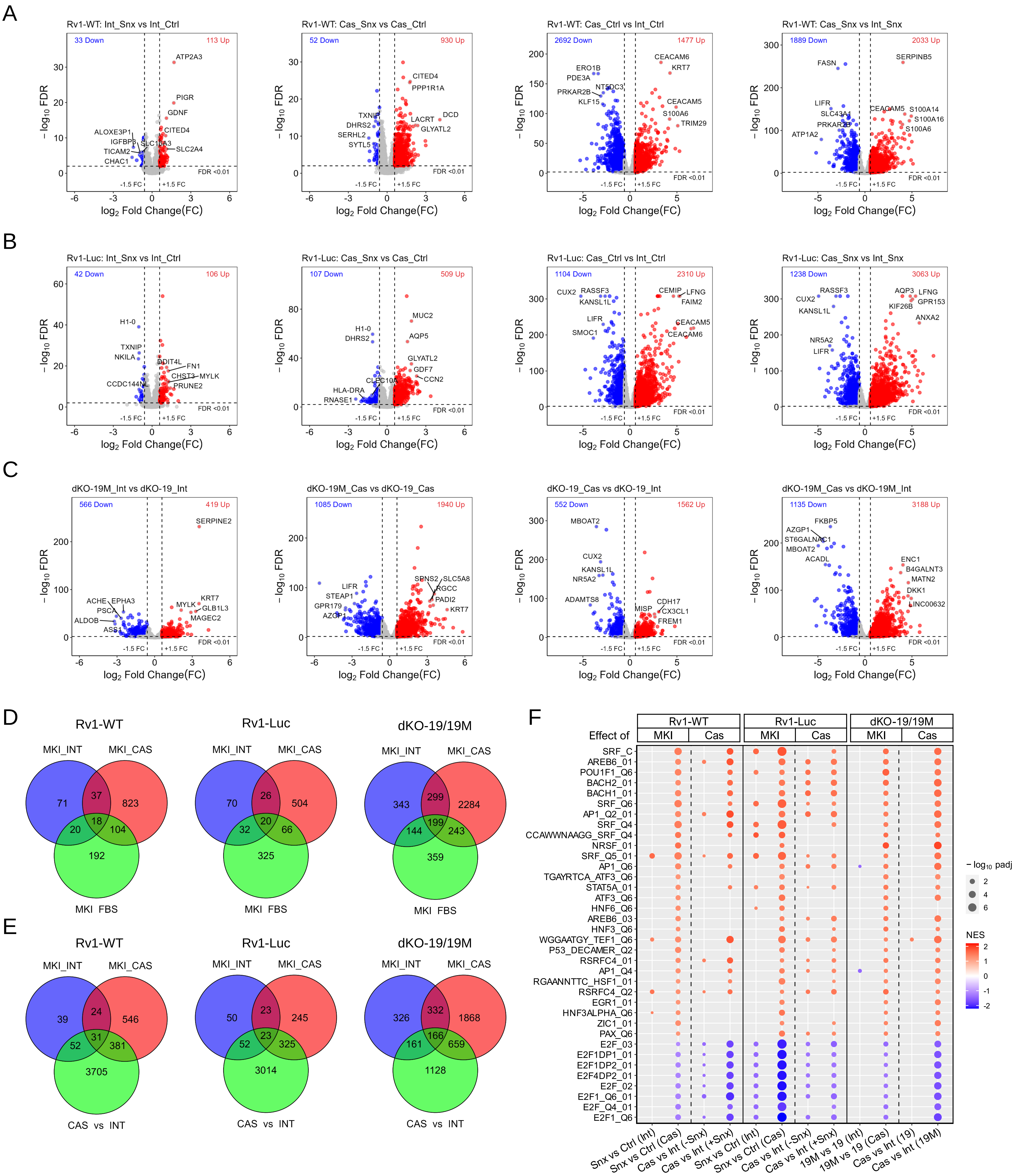

Figure S2. RNA-Seq analysis of the effects of CDK8/19 inhibition in 22Rv1 derivatives *in vivo* on the expression of tumor (human) genes.

(A-C) Effects of Mediator kinase inhibition (MKI) (SNX631 vs Ctrl or dKO-19M vs dKO-19) in intact or castrated NSG mice and the effects of castration in the absence or presence of MKI for Rv1-WT **(A)**, Rv1-Luc **(B)** and dKO-19/19M **(C)** tumors *in vivo*. **(D)** Overlap of tumor DEGs affected by MKI *in vitro* (FBS), in intact (INT) and castrated (CAS) animals for Rv1-WT, Rv1-Luc and dKO-19/19M models. **(E)** Overlap of tumor DEGs affected by MKI or castration (CAS) in intact (INT) and castrated (CAS) animals for Rv1-WT, Rv1-Luc and dKO-19/19M models. **(F)** Effects of MKI and of castration (Cas) under the indicated conditions on the pathways affected by Mediator kinase inhibition in three 22Rv1 tumor models in castrated animals. Affected pathways were identified by GSEA of C3 transcription factor targets signature gene sets (MSigDB Human collections).

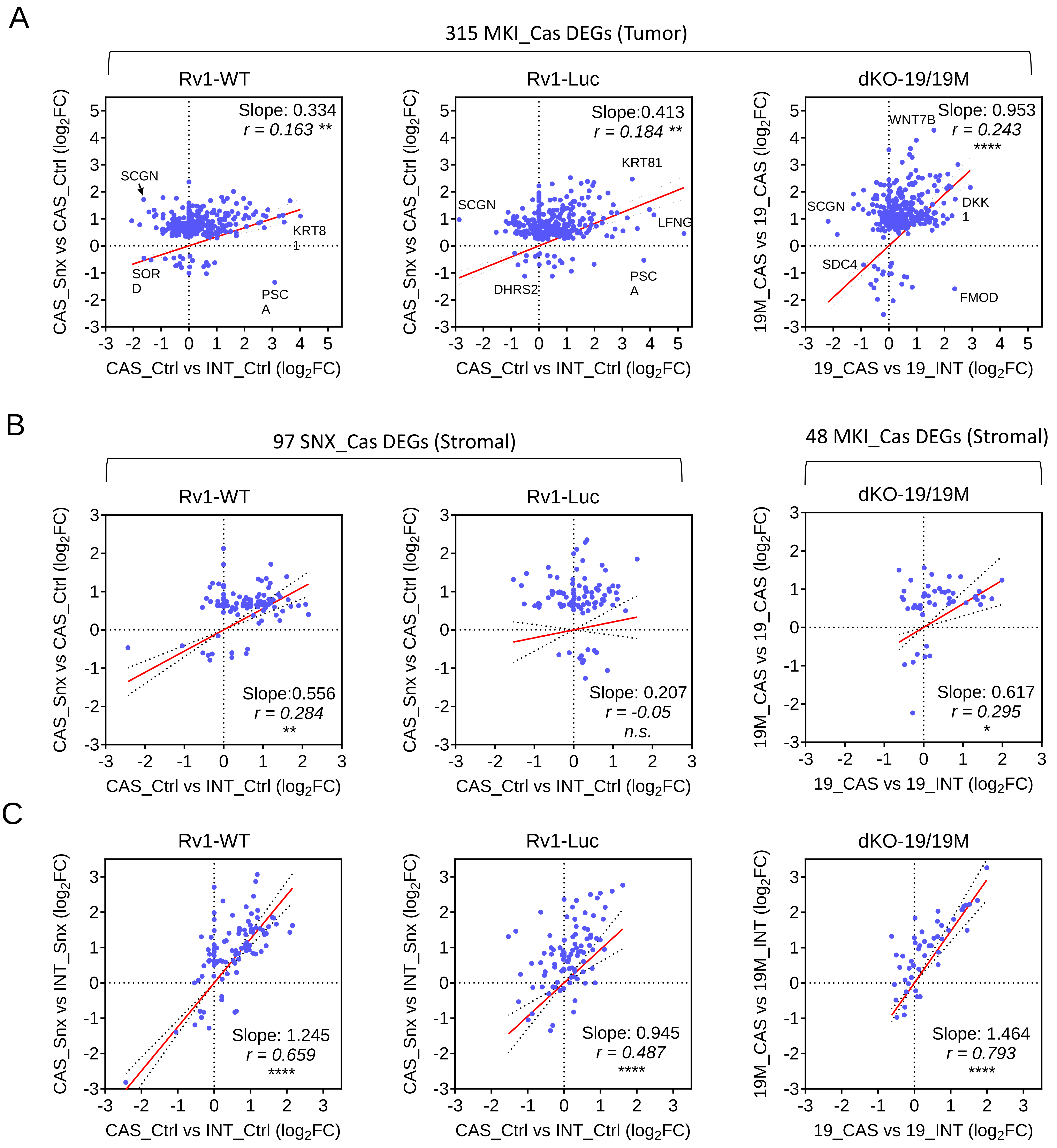

Figure S3. Correlation of the transcriptomic effects of Mediator kinase inhibition (MKI) and castration on tumor and stromal DEGs affected by MKI in 22Rv1 xenografts.

(A) Correlation of the effects of MKI in castrated animals and the effects of castration on the 315 tumor (human) DEGs co-regulated by MKI in three 22Rv1 models. (**, p<0.01; ****, p<0.0001). **(B)** Correlation of the effects of MKI in castrated animals and the effects of castration on stromal (mouse) DEGs regulated by Mediator kinase mutagenesis and/or SNX631 treatment. (n.s., not significant; *, p<0.05; **, p<0.01). **(C)** Correlation of the effects of castration with and without MKI on stromal DEGs regulated by Mediator kinase mutagenesis and/or SNX631 treatment. (****, p<0.0001).

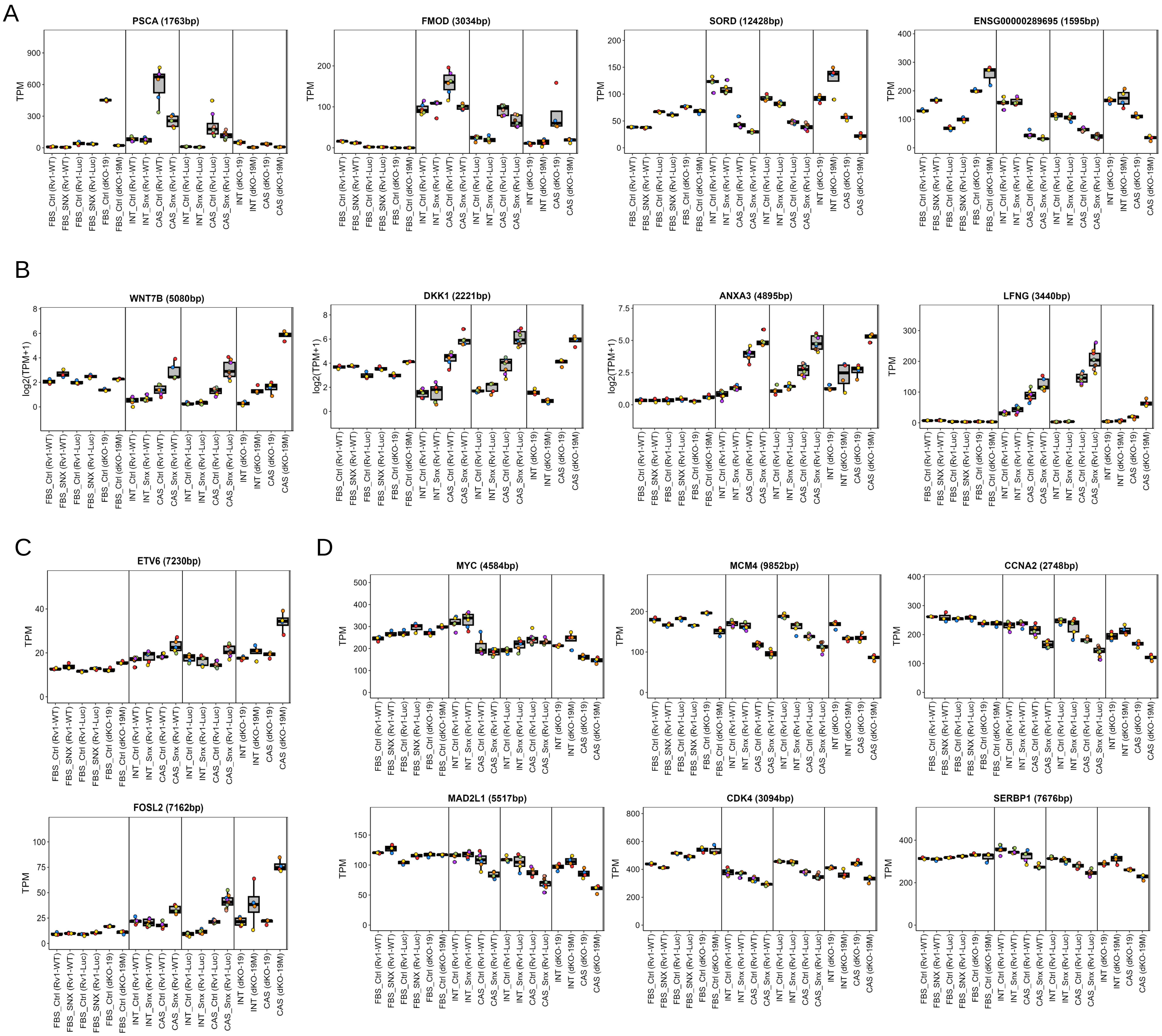

Figure S4. Expression of representative Mediator kinase-regulated tumor (human) genes under different conditions *in vitro* and *in vivo* (RNA-Seq).

(A) DEGs negatively regulated by MKI in 22Rv1 xenografts growing in castrated animals. **(B)** DEGs positively regulated by MKI in 22Rv1 xenografts growing in castrated animals. **(C)** ETV6 and FOSL2 genes. **(D)** MYC and its targets MCM4, CCNA2, MAD2L1, CDK4 and SERBP1.

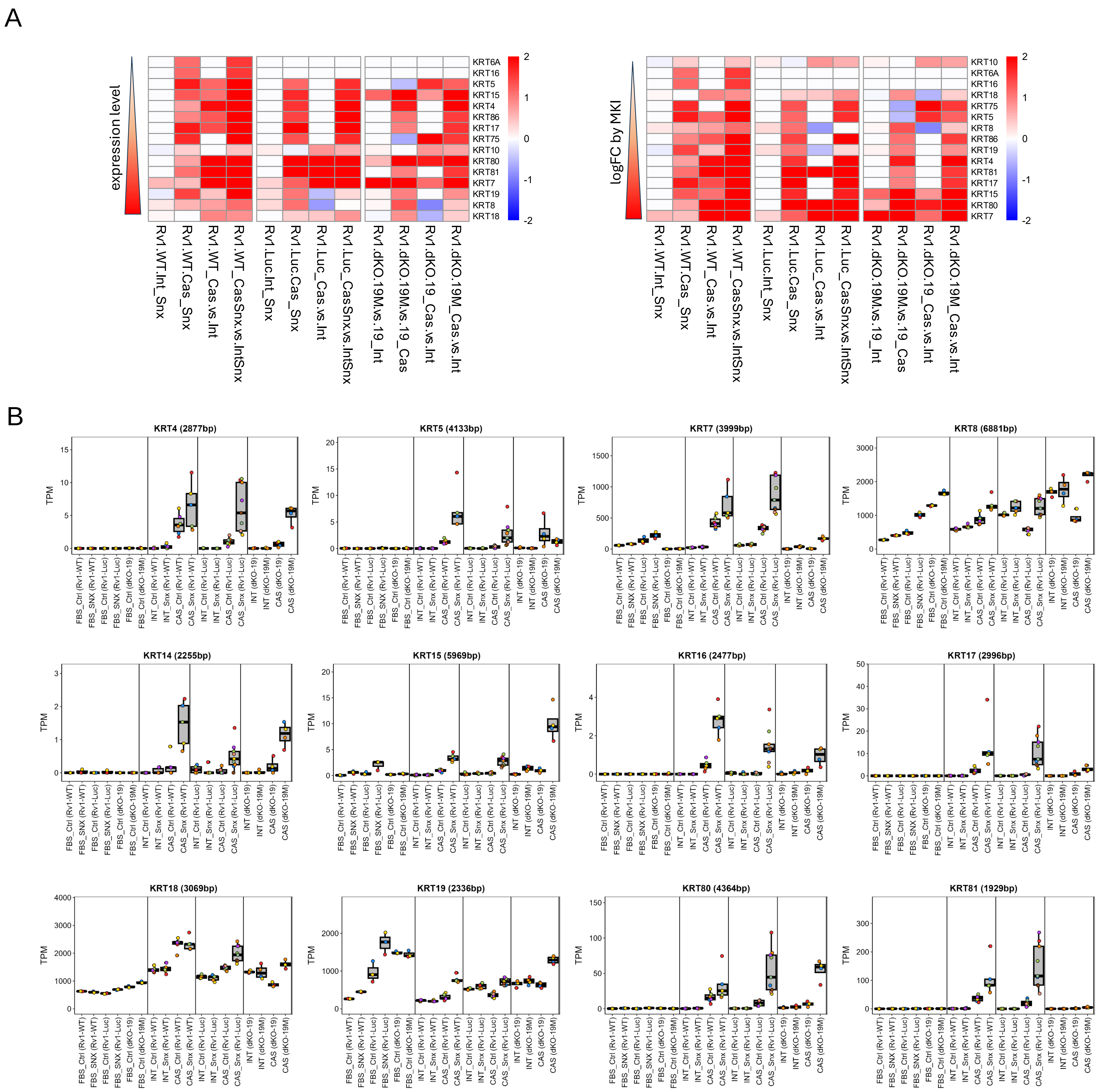

Figure S5. Expression of keratin family genes in 22Rv1 xenografts under different conditions *in vitro* and *in vivo*

(A) Heatmap of 16 keratin family genes expressed in 22Rv1 in the indicated comparisons (left: ordered by expression level; right: order by logFC of MKI under castration condition). **(B)** Expression of keratin genes under different conditions *in vitro* and *in vivo* (RNA-Seq).

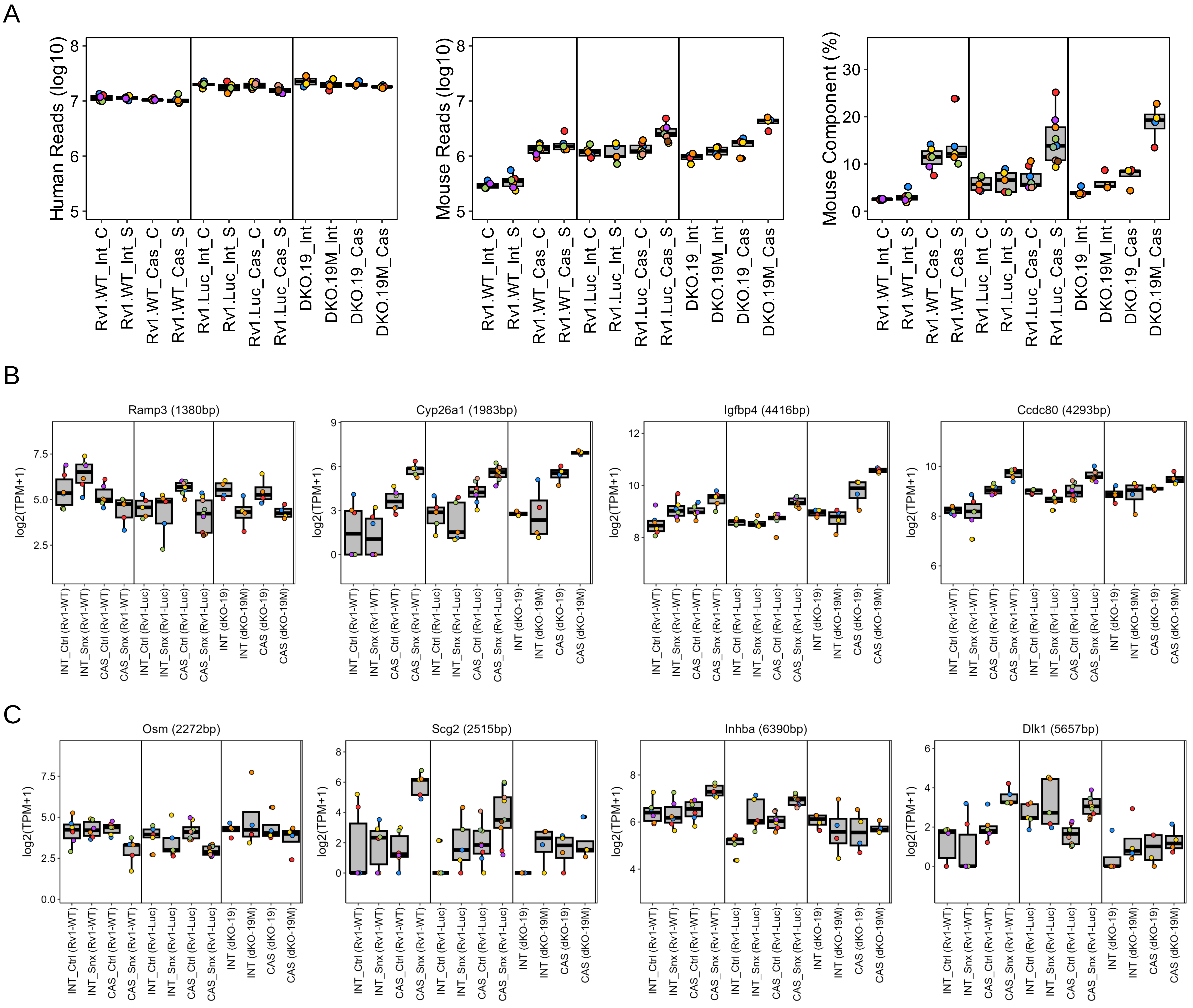

Figure S6. RNA-Seq analysis of the effects of Mediator kinase inhibition in 22Rv1 tumors on the expression of stromal (mouse) genes.

(A) Total human gene counts (left), total mouse gene counts (middle) and the percentage of mouse gene counts (right) in the indicated tumor samples. **(B,C)** Expression of representative stromal DEGs negatively or positively regulated by SNX631 treatment and Mediator kinase Mediator kinase mutagenesis **(B)** or by SNX631 treatment alone **(C)** in 22Rv1 xenografts growing in castrated animals.

A

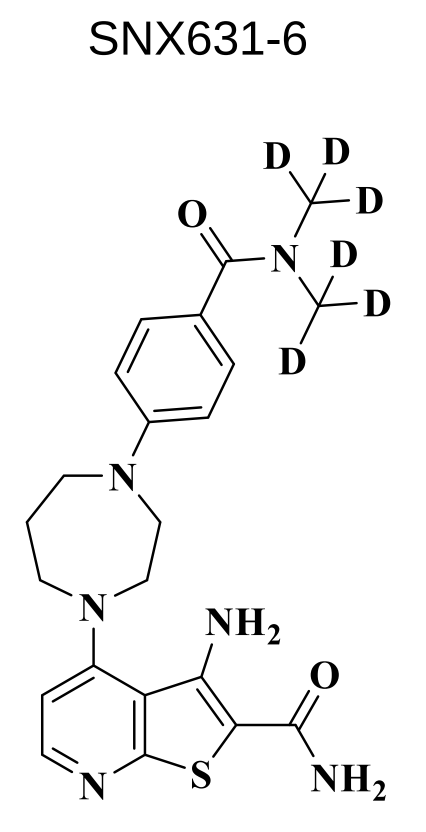

B

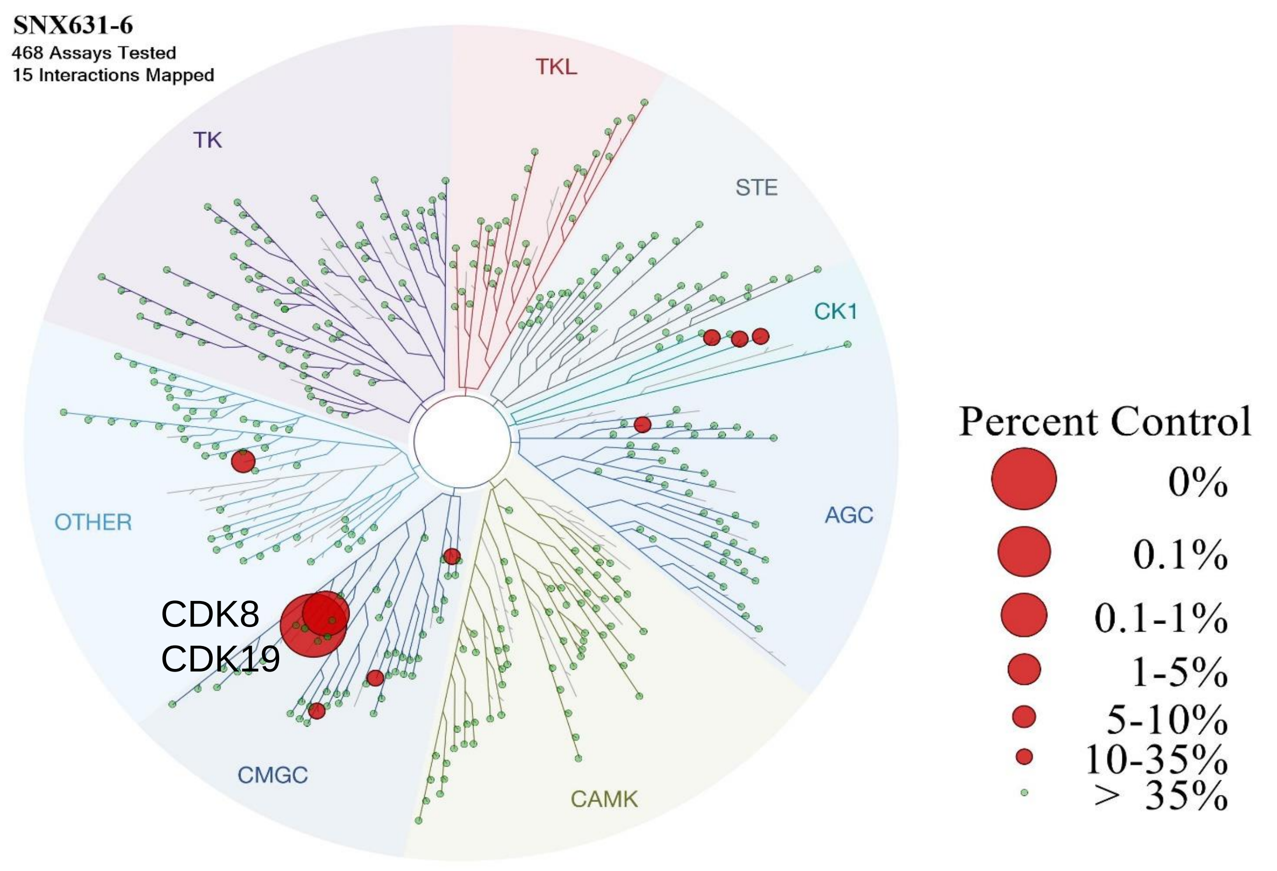

C

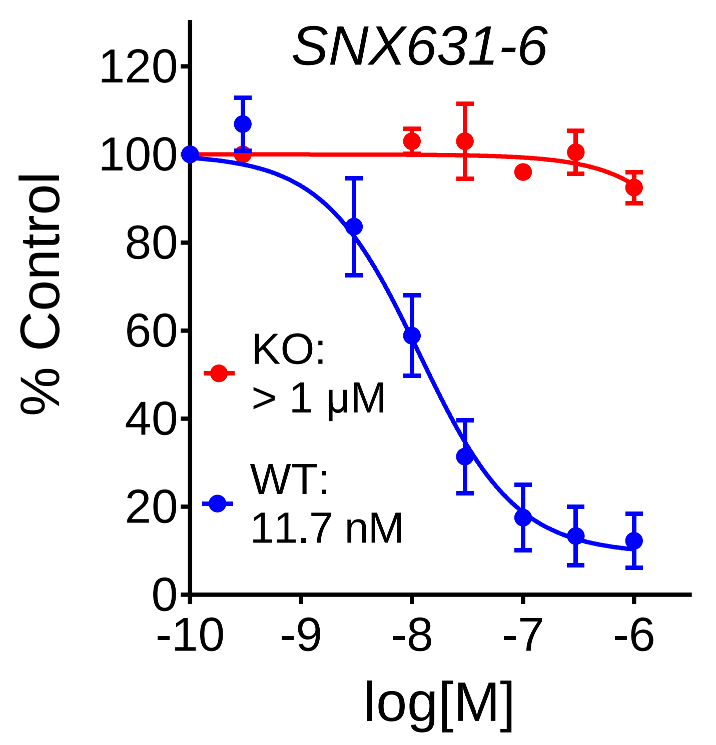

D

In vitro KINETICfinder Assay (Enzymlogic)

Client_ID	Target	k_{on} ($M^{-1}s^{-1}$)	k_{off} (s^{-1})	t (min)	$T_{1/2}$ (min)	K_d (M)
Senexin B	CDK8/cyclin C	1.65E+06	3.25E-03	5.1	3.6	1.97E-09
SNX631	CDK8/cyclin C	1.05E+06	2.83E-04	59	41	2.70E-10
SNX631-6	CDK8/cyclin C	1.49E+06	3.72E-04	45	31	2.50E-10
Staurosporine	CDK8/cyclin C	1.12E+05	5.55E-04	30	21	4.95E-09
Senexin B	CDK19/cyclin C	2.13E+06	6.28E-03	2.7	1.8	2.95E-09
SNX631	CDK19/cyclin C	1.12E+06	3.56E-04	47	32	3.19E-10
SNX631-6	CDK19/cyclin C	9.79E+05	3.03E-04	55	38	3.09E-10
Staurosporine	CDK19/cyclin C	1.07E+05	2.45E-04	68	47	2.30E-09

Figure S7. Characteristics of CDK8/19 inhibitor SNX631-6.

(A) Chemical structure of SNX631-6. **(B)** Kinome profiling (Discover X, 468 kinases) of SNX631-6 at 2 µM. **(C)** Effects of SNX631-6 in a cell-based NFκB-dependent reporter assay for CDK8/19 inhibition in wild type (WT) and CDK8/19-double knockout (KO) 293 cells. **(D)** Binding kinetics of Senexin B, SNX631, SNX631-6 and Staurosporine to recombinant CDK8/CycC and CDK19/CycC proteins in the KINETICfinder Assay (Enzymlogic).

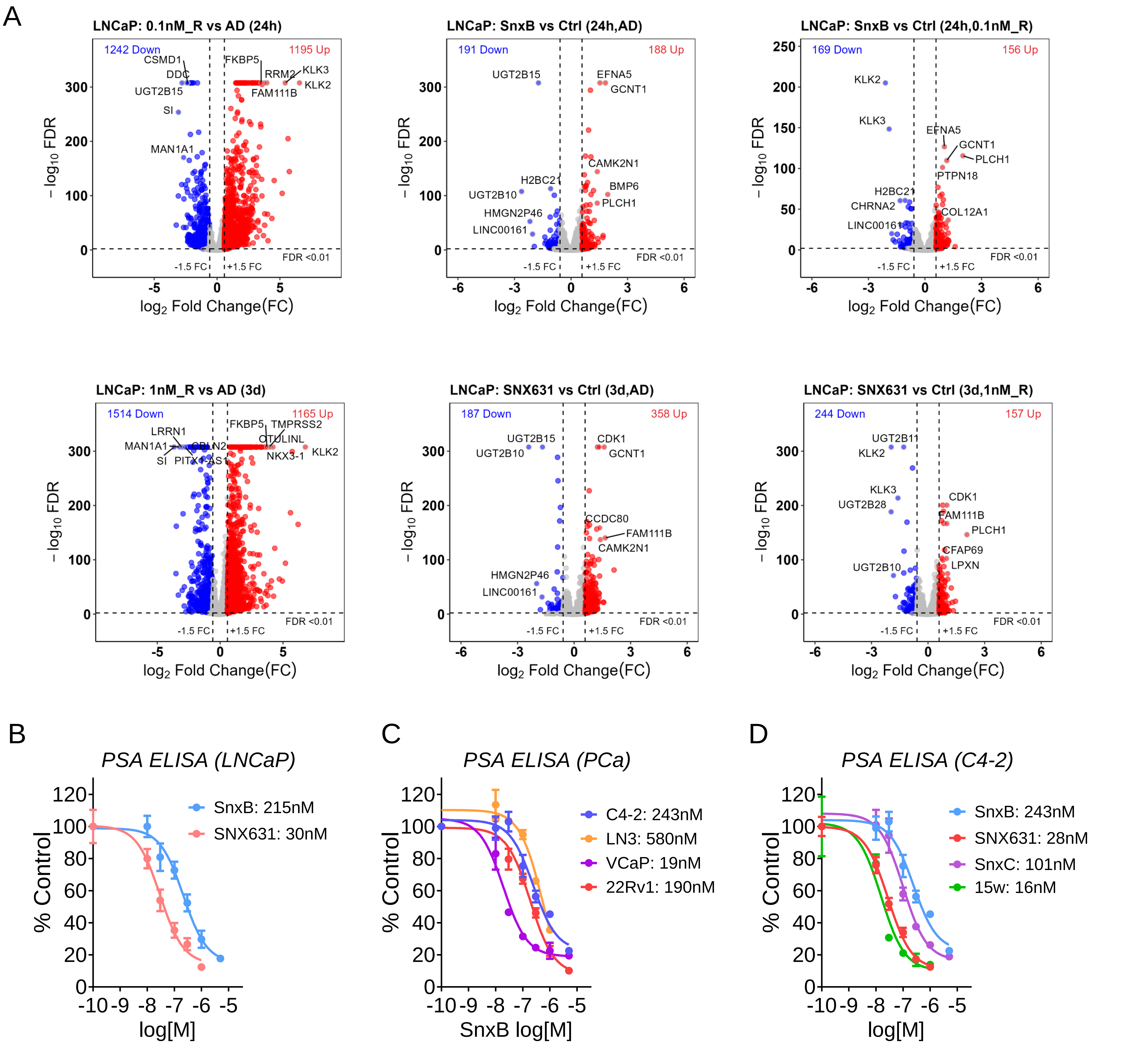

Figure S8. Effects of CDK8/19 inhibition on gene expression in AR-positive PCa cells *in vitro*.

(A) RNA-Seq analysis of the effects of androgen stimulation and CDK8/19 inhibition by Senexin B or SNX631 in LNCaP cells. Differentially expressed genes (DEGs) are marked with red (upregulated) and blue (downregulated) dots. **(B)** Effects of Senexin B and SNX631 treatment (4 d) on PSA protein expression (ELISA) in conditioned media of LNCaP cells in FBS media. **(C)** Effects of Senexin B treatment on PSA expression in conditioned media of different AR-positive PCa cell lines. **(D)** Effects of different CDK8/19 inhibitors on PSA expression in conditioned media of C4-2 cells.

A

PDX_ID	Tissue CEA+	Tissue PSA+	Prior Rx	Primary explant
SM0310	Neg	Pos	Lupron, Casodex, abiraterone, docetaxel	skin metastasis
CG0509	Neg	Pos	Lupron, docetaxel, carboplatin	prostatectomy

B

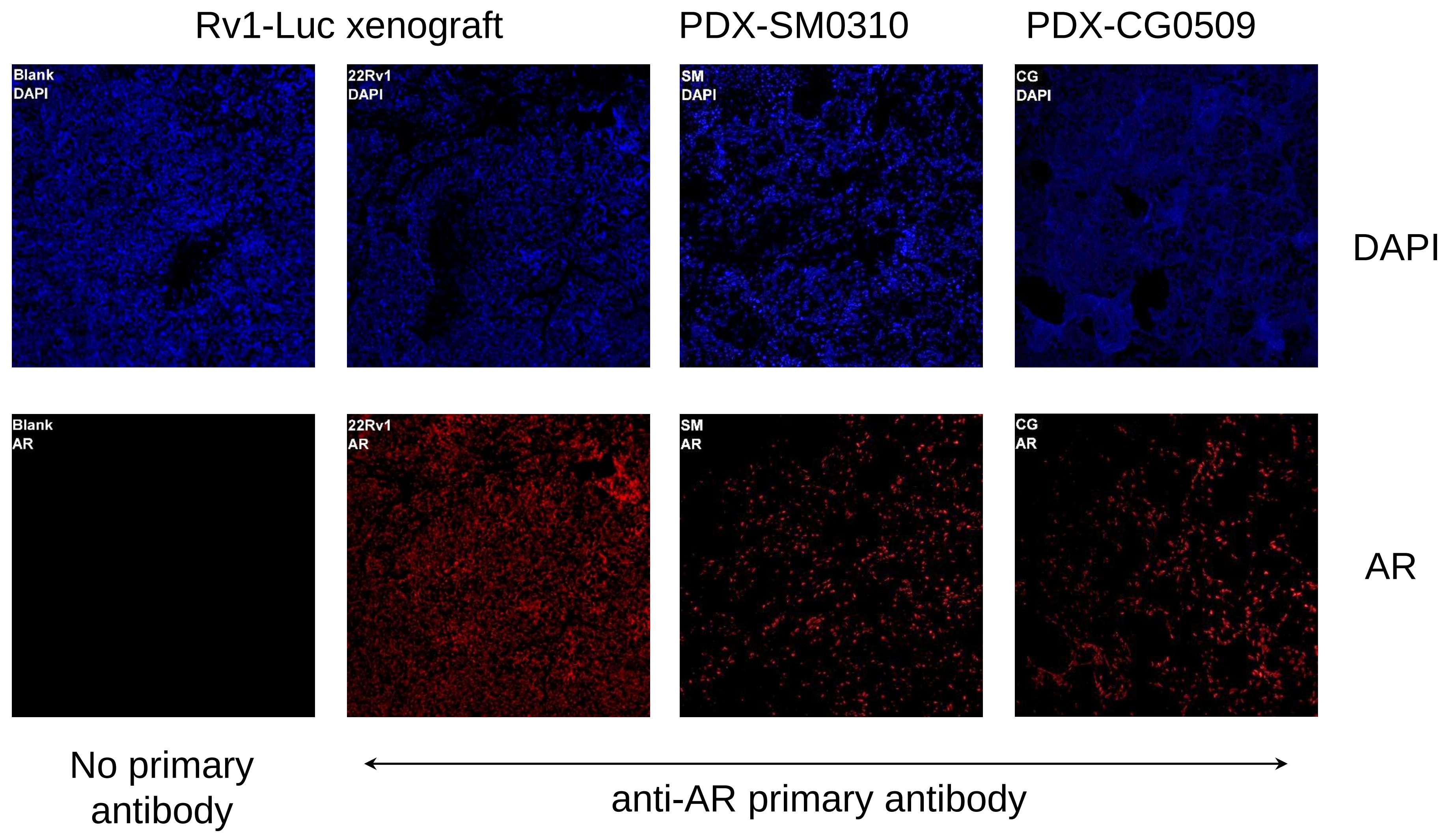

C

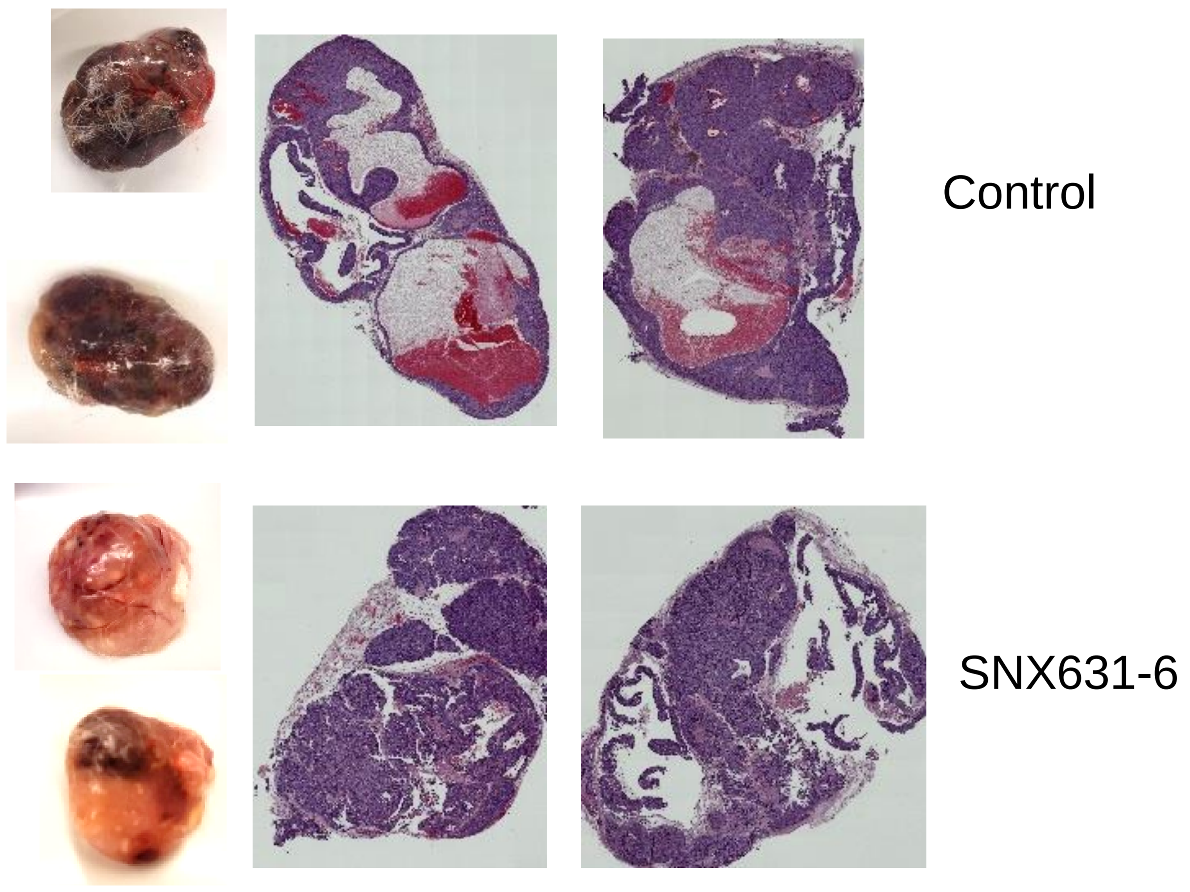

Figure S9. Characteristics of PDX models and effects of SNX631-6 on tumor vasculature.

(A) Clinical background on SM0310 and CG0509 PDXs. **(B)** Immunostaining of tissue sections from Rv1-Luc, SM0310 and CG0509 xenografts for AR and DAPI. **(C)** Macroscopic images (left) and H&E staining (right) of control and SNX631-6-treated J000077451 PDX tumors.

Supplementary Tables

Table S5. List of Key Resources

REAGENT or MATERIALS	SOURCE	IDENTIFIER
**Antibodies**		
Anti-CDK8 (goat polyclonal)	Santa Cruz Biotechnology	Cat# sc-1521; RRID:AB_2260300
Anti-CDK19 (goat polyclonal)	Chen et. al. 2023[1]	N/A
Anti-AR (rabbit polyclonal)	Santa Cruz Biotechnology	Cat# sc-13062; RRID:AB_633881
Anti-Phospho-STAT1 (Ser727)	Cell Signaling Technology	Cat# 8826S; RRID:AB_2773718
Anti-STAT1	Santa Cruz Biotechnology	Cat# sc-592; RRID:AB_632434
Anti-GAPDH	Santa Cruz Biotechnology	Cat# sc-32233; RRID:AB_627679
Donkey anti-goat IgG-HRP	Santa Cruz Biotechnology	Cat# sc-2020; RRID:AB_631728
Mouse anti-goat IgG-HRP	Santa Cruz Biotechnology	Cat# sc-2354; RRID:AB_628490
Peroxidase-linked sheep anti-mouse IgG	GE Healthcare	Cat# NXA931; RRID:AB_772209
Peroxidase-linked donkey anti-rabbit IgG	GE Healthcare	Cat# NA934; RRID:AB_772206
Donkey anti-Rabbit IgG (H+L) Highly Cross-Adsorbed Secondary Antibody, Alexa Fluor™ 555	Thermo Fisher Scientific	Cat# A-31572; RRID:AB_162543
**Bacterial and virus Strains**		
NEB® Stable Competent E. coli (High Efficiency)	New England Biolabs	Cat# C3040I
**Chemicals, peptides, and recombinant Proteins**		
Senexin B	Senex Biotechnology	N/A
Senexin C	Senex Biotechnology	N/A
SNX631	Senex Biotechnology	N/A
SNX631-6	Senex Biotechnology	N/A
15w	Li et al. 2019[2]	N/A
R1881	Sigma-Aldrich	Cat# R0908
Enzalutamide (MDV3100)	Sigma-Aldrich	Cat# PHB00235
Degarelix (Firmagon® 80 mg)	Ferring Pharmaceuticals	NDC 55566-8303-1
Puromycin dihydrochloride	Tocris Bioscience	Cat# 408950
Blasticidin S, Hydrochloride	MilliporeSigma™	Cat# 20335025MG
Halt Protease Inhibitor Cocktail	Thermo Scientific™	Cat# 78438
D-MEM (high-glucose)	Cytiva	Cat# SH30022.01
RPMI-1640	Cytiva / Hyclone	Cat# SH30027.01
EMEM	ATCC	Cat# 30-2003™
Fetal Bovine Serum (FBS)	Cytiva / Hyclone	Cat# SH30396.03
Charcoal Stripped FBS (CSS)	Gibco	Cat# 12676029

L-Glutamine, 200 mM	Cytiva / Hyclone	Cat# SH30034.01
Sodium Pyruvate, 100 mM	Cytiva / Hyclone	Cat# SH30239.01
Sodium Bicarbonate, 7.5%	Cytiva / Hyclone	Cat# SH30033.01
HEPES, 1 M , pH 7.4	Cytiva / Hyclone	Cat# SH30237.01
D-(+)-Glucose solution, 45% in H_2O	Sigma-Aldrich	Cat# G8769
Penicillin/Streptomycin/Glutamine (100x)	Cytiva / Hyclone	Cat# SV30010
Corning™ Matrigel™ Membrane Matrix	Fisher Scientific	Cat# CB-40234
**Critical Commercial Assays**		
MycoAlert™ Mycoplasma Detection Kit	Lonza	Cat# LT07
DC Protein Assay Kit	Bio-Rad Laboratories	Cat# 5000116
RNeasy Mini Kit	QIAGEN	Cat# 74106
RNAlater stabilization solution	Thermo Fisher Scientific	Cat# AM7020
miRNeasy Mini Kit	QIAGEN	Cat# 74106
PSA ELISA kit	Abcam	Cat# ab113327
iScript cDNA synthesis kit	Bio-Rad Laboratories	Cat# 1708891
qScript Ultra SuperMix	QuantaBio	Cat# 95217
iTaq™ Universal SYBR® Green Supermix	Bio-Rad Laboratories	Cat# 172-5124
PerfeCTa SYBR Green FastMix	QuantaBio	Cat# 95072
NEBNext Ultra II Directional RNA Library Prep Kit	New England Biolabs	Cat# E7760
Plasmid mini prep Kit	QIAGEN	Cat# 2710
QiaQuick Gel Extraction Kit	QIAGEN	Cat# 28706
CloneDirect™ Rapid Ligation Kit	Lucigen	Cat# 40020
Phusion Site-Directed Mutagenesis Kit	Thermo Scientific	Cat# F541
Western Lightning® Plus-ECL reagent	PerkinElmer Inc.	Cat# NEL105001EA
**Experimental Models: Cell Lines**		
HEK293 (293)	ATCC	CRL-1573; RRID:CVCL_0045
293-dKO	Li et al. 2019[2]	N/A
LNCaP	ATCC	CRL-1740
LNCaP-C4-2	ATCC	CRL-3314
LNCaP-LN3	Gift from Dr. Isaiah Fidler'lab	N/A
VCaP	ATCC	CRL-2876
DU145	ATCC	HTB-81
PC-3	ATCC	CRL-1435
MYC-CaP-CR	Ellis L et. al. 2012[3]	N/A
22Rv1	ATCC	CRL-2505; RRID:CVCL_1045
Rv1-dKO	Chen et. al. 2023[1]	N/A
Rv1-Luc (Rv1 derivatives expressing luciferase)	This study	N/A

Rv1-dKO-V (empty expressing vector)	This study	N/A
Rv1-dKO-19 (expressing CDK19-WT)	This study	N/A
Rv1-dKO-19M (expressing CDK19-D173A)	This study	N/A
Rv1-dKO-8 (expressing CDK8-WT)	This study	N/A
Rv1-dKO-8M (expressing CDK8-D173A)	This study	N/A
Rv1-dKO-19-8 (expressing CDK19-WT and CDK8-WT)	This study	N/A
Rv1-dKO-19-8M (expressing CDK19-WT and CDK8-D173A)	This study	N/A
Rv1-dKO-19M-8 (expressing CDK19-D173A and CDK8-WT)	This study	N/A
Rv1-dKO-19M-8M (expressing CDK19-D173A and CDK8-D173A)	This study	N/A
**Recombinant DNA: Vectors and Oligonucleotides**		
PCR/Cloning primers and guide RNAs	This paper	Table S6
pHIV-dTomato	Chen et. al. 2023[1]	RRID:Addgene_21374
pHIV-Luc-ZsGreen	Chen et. al. 2023[1]	RRID:Addgene_39196
pHIV-dTomato-CDK8	Chen et. al. 2023[1]	N/A
pHIV-dTomato-CDK8M	Chen et. al. 2023[1]	N/A
pHIV-dTomato-CDK19	Chen et. al. 2023[1]	N/A
pHIV-dTomato-CDK19M	Chen et. al. 2023[1]	N/A
pHIV-Luc-BlastR	Chen et. al. 2023[1]	N/A
pHIV-dTomato-CDK19_CasR	This paper	N/A
pHIV-dTomato-CDK19M_CasR	This paper	N/A
pHIV-ZsGreen-CDK8	This paper	N/A
pHIV-ZsGreen-CDK8M	This paper	N/A
pHIV-ZsGreen-CDK8_CasR	This paper	N/A
pHIV-ZsGreen-CDK8M_CasR	This paper	N/A
**Software and Algorithms**		
GraphPad Prism 9	Graph Pad Software	RRID:SCR_002798
Image Lab version 5.2.1 build 11	Bio-Rad	RRID:SCR_014210
Bio-Rad CFX Manager 3.0	Bio-Rad	Bio-Rad CFX Manager 3.0
Cutadapt version 2.8	Marcel et al. 2011[4]	RRID:SCR_011841
STAR version 2.7.2b	Dobin et al. 2013[5]	RRID:SCR_004463
featureCount / Subread v2.0.3	Liao et al. 2019[6]	RRID:SCR_009803
R version 3.6.1	R Core Team[7]	https://www.R-project.org/
DeSeq2 package	Michael et al. 2014[8]	RRID:SCR_015687
fgsea	Korotkevich et al. 2016[9]	RRID:SCR_020938
tidyverse	Wickham et al. 2019[10]	RRID:SCR_019186
pheatmap	Kolde R. 2015[11]	RRID:SCR_016418

TCGAbiolinks	Antonio et al. 2015[12]	RRID:SCR_017683
ggvenn	Yan L. 2023[13]	N/A
ggcor	Huang et al. 2020[14]	N/A
cBioportalData	Ramos et al. 2020[15]	N/A
survminer	Kassambara A. 2021[16]	RRID:SCR_021094

References

1 Chen, M. *et al.* CDK8 and CDK19: positive regulators of signal-induced transcription and negative regulators of Mediator complex proteins. *Nucleic Acids Research* (2023). https://doi.org:10.1093/nar/gkad538

2 Li, J. *et al.* Characterizing CDK8/19 Inhibitors through a NFκB-Dependent Cell-Based Assay. *Cells* **8**, 1208 (2019).

3 Ellis, L., Lehet, K., Ramakrishnan, S., Adelaiye, R. & Pili, R. Development of a castrate resistant transplant tumor model of prostate cancer. *Prostate* **72**, 587-591 (2012). https://doi.org:10.1002/pros.21465

4 Martin, M. Cutadapt removes adapter sequences from high-throughput sequencing reads. *2011* **17**, 3 (2011). https://doi.org:10.14806/ej.17.1.200

5 Dobin, A. *et al.* STAR: ultrafast universal RNA-seq aligner. *Bioinformatics* **29**, 15-21 (2013). https://doi.org:10.1093/bioinformatics/bts635

6 Liao, Y., Smyth, G. K. & Shi, W. The R package Rsubread is easier, faster, cheaper and better for alignment and quantification of RNA sequencing reads. *Nucleic Acids Research* **47**, e47-e47 (2019). https://doi.org:10.1093/nar/gkz114

7 Team, R. C. R: A language and environment for statistical computing. 2014. *R Foundation for Statistical Computing: Vienna, Austria* (2018).

8 Love, M. I., Huber, W. & Anders, S. Moderated estimation of fold change and dispersion for RNA-seq data with DESeq2. *Genome Biology* **15**, 550 (2014). https://doi.org:10.1186/s13059-014-0550-8

9 Wu, T. *et al.* clusterProfiler 4.0: A universal enrichment tool for interpreting omics data. *The Innovation* **2**, 100141 (2021). https://doi.org:https://doi.org/10.1016/j.xinn.2021.100141

10 Wickham, H. *et al.* Welcome to the Tidyverse. *Journal of open source software* **4**, 1686 (2019).

11 Kolde, R. & Kolde, M. R. Package 'pheatmap'. *R package* **1**, 790 (2015).

12 Colaprico, A. *et al.* TCGAbiolinks: an R/Bioconductor package for integrative analysis of TCGA data. *Nucleic Acids Research* **44**, e71-e71 (2015). https://doi.org:10.1093/nar/gkv1507

13 Yan, L. ggvenn: Draw Venn Diagram by 'ggplot2'. (2023).

14 Houyun Huang, L. Z., Jian Chen, Taiyun Wei. ggcor: Extended tools for correlation analysis and visualization. (2020).

15 Ramos, M. *et al.* Multiomic Integration of Public Oncology Databases in Bioconductor. *JCO Clin Cancer Inform* **4**, 958-971 (2020). https://doi.org:10.1200/cci.19.00119

16 Alboukadel Kassambara, M. K., Przemyslaw Biecek. survminer: Drawing Survival Curves using 'ggplot2'. (2021).
