# Supplementary material for: Mediator kinase inhibition reverses castration resistance of advanced prostate cancer
Source: J Clin Invest. 2024 Mar 28;134(10):e176709. doi: 10.1172/JCI176709 (PMC11093614; doi:10.1172/JCI176709)
Supplement: Unedited blot and gel images [file jci-134-176709-s024.pptx]

## Slide 1
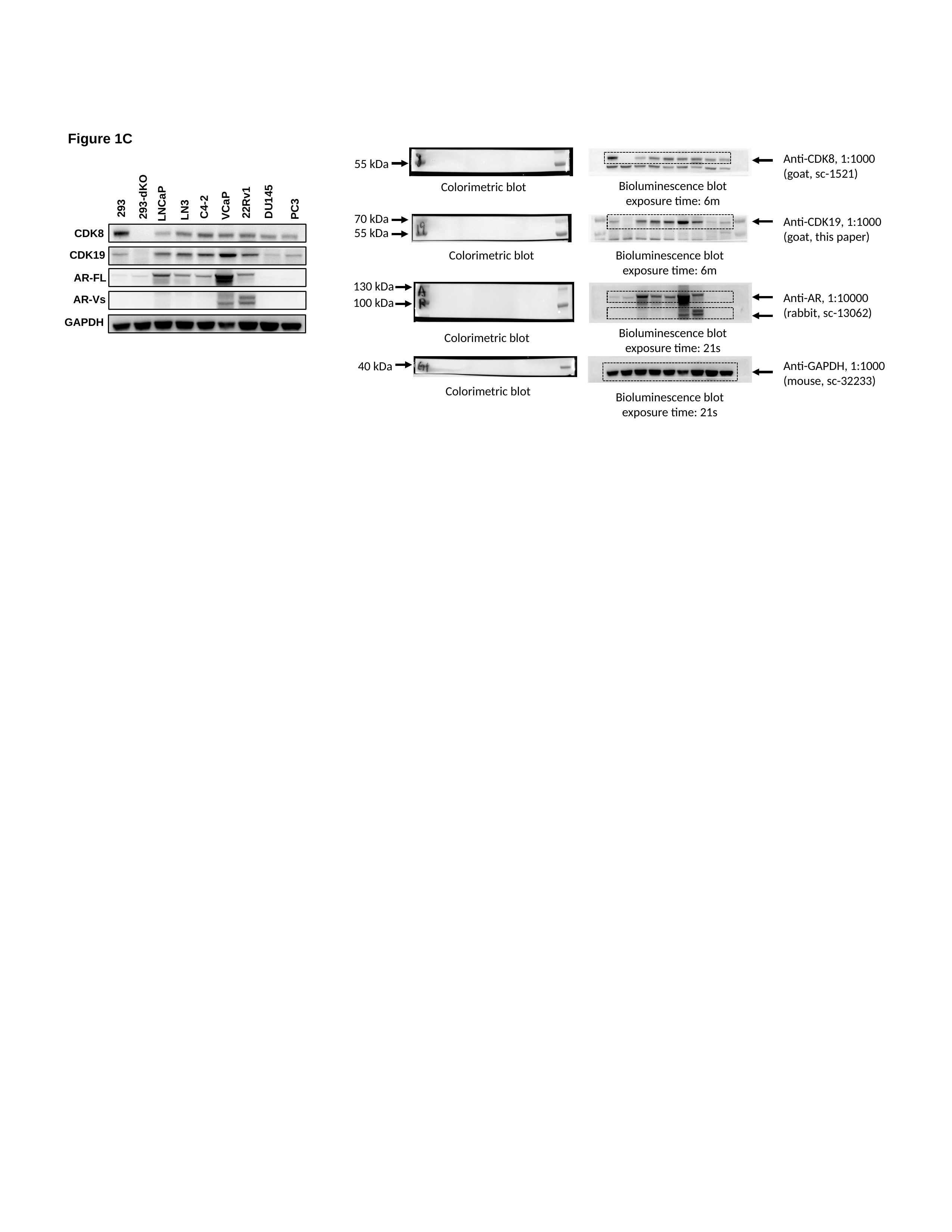

Figure 1C
Anti-CDK8, 1:1000
(goat, sc-1521)
55 kDa
293-dKO
DU145
22Rv1
LNCaP
VCaP
C4-2
293
PC3
LN3
CDK8
CDK19
AR-FL
AR-Vs
GAPDH
Bioluminescence blot
exposure time: 6m
Colorimetric blot
70 kDa
Anti-CDK19, 1:1000
(goat, this paper)
55 kDa
Colorimetric blot
Bioluminescence blot
exposure time: 6m
130 kDa
Anti-AR, 1:10000
(rabbit, sc-13062)
100 kDa
Bioluminescence blot
exposure time: 21s
Colorimetric blot
Anti-GAPDH, 1:1000
(mouse, sc-32233)
40 kDa
Colorimetric blot
Bioluminescence blot
exposure time: 21s

## Slide 2
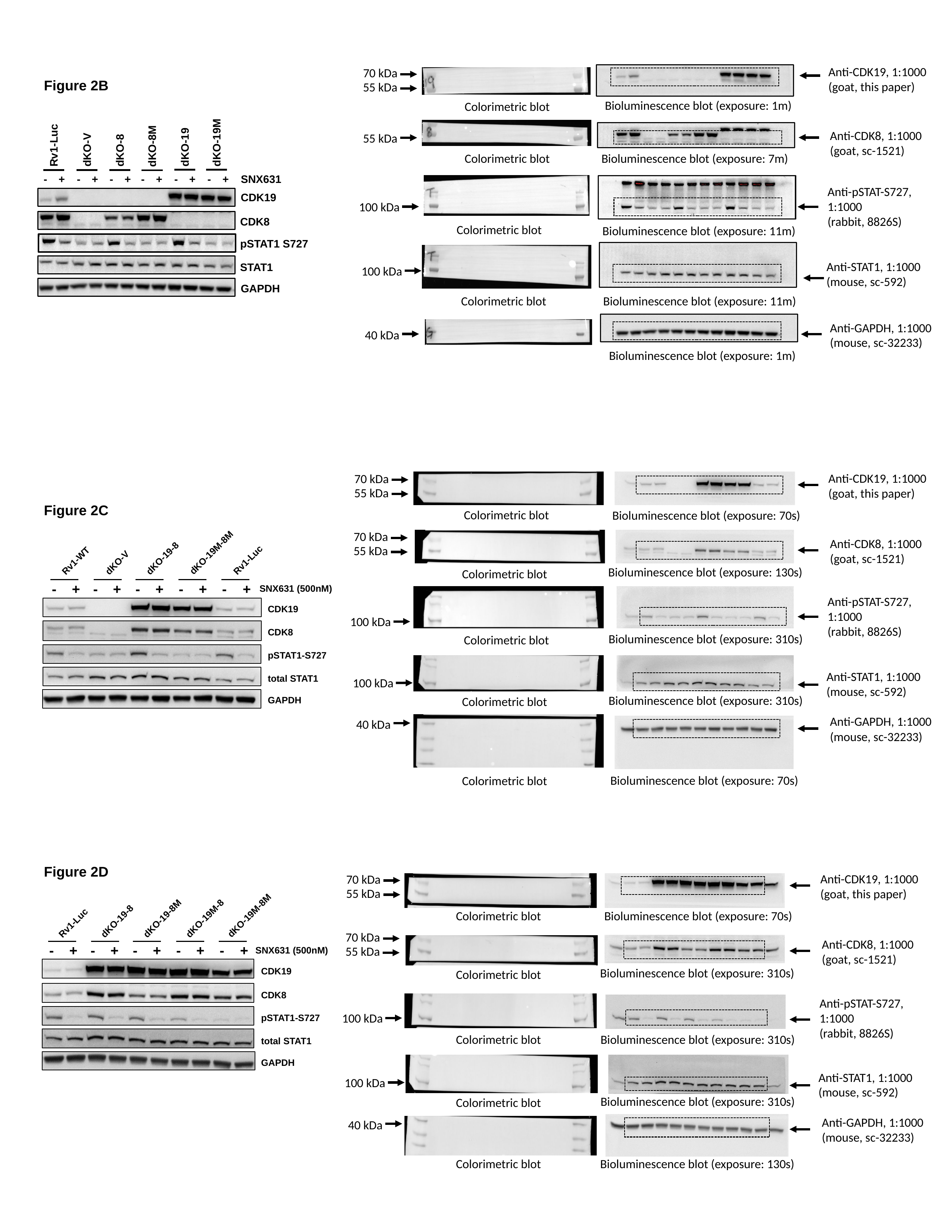

Anti-CDK19, 1:1000
(goat, this paper)
70 kDa
55 kDa
Bioluminescence blot (exposure: 1m)
Colorimetric blot
Anti-CDK8, 1:1000
(goat, sc-1521)
55 kDa
Colorimetric blot
Bioluminescence blot (exposure: 7m)
Anti-pSTAT-S727, 1:1000
(rabbit, 8826S)
100 kDa
Colorimetric blot
Bioluminescence blot (exposure: 11m)
Anti-STAT1, 1:1000
(mouse, sc-592)
100 kDa
Bioluminescence blot (exposure: 11m)
Colorimetric blot
Anti-GAPDH, 1:1000
(mouse, sc-32233)
40 kDa
Bioluminescence blot (exposure: 1m)
Figure 2B
dKO-19
dKO-19M
dKO-V
dKO-8
dKO-8M
Rv1-Luc
-
+
-
+
-
+
-
+
-
+
-
+
SNX631
CDK19
CDK8
pSTAT1 S727
STAT1
GAPDH
Anti-CDK19, 1:1000
(goat, this paper)
70 kDa
55 kDa
Colorimetric blot
Bioluminescence blot (exposure: 70s)
70 kDa
Anti-CDK8, 1:1000
(goat, sc-1521)
55 kDa
Bioluminescence blot (exposure: 130s)
Colorimetric blot
Anti-pSTAT-S727, 1:1000
(rabbit, 8826S)
100 kDa
Bioluminescence blot (exposure: 310s)
Colorimetric blot
Anti-STAT1, 1:1000
(mouse, sc-592)
100 kDa
Bioluminescence blot (exposure: 310s)
Colorimetric blot
Anti-GAPDH, 1:1000
(mouse, sc-32233)
40 kDa
Bioluminescence blot (exposure: 70s)
Colorimetric blot
Figure 2C
dKO-19M-8M
dKO-19-8
Rv1-Luc
Rv1-WT
dKO-V
-
+
-
+
-
+
-
+
-
+
SNX631 (500nM)
CDK19
CDK8
pSTAT1-S727
total STAT1
GAPDH
Figure 2D
Anti-CDK19, 1:1000
(goat, this paper)
70 kDa
55 kDa
Colorimetric blot
Bioluminescence blot (exposure: 70s)
70 kDa
Anti-CDK8, 1:1000
(goat, sc-1521)
55 kDa
Bioluminescence blot (exposure: 310s)
Colorimetric blot
Anti-pSTAT-S727, 1:1000
(rabbit, 8826S)
100 kDa
Colorimetric blot
Bioluminescence blot (exposure: 310s)
Anti-STAT1, 1:1000
(mouse, sc-592)
100 kDa
Bioluminescence blot (exposure: 310s)
Colorimetric blot
Anti-GAPDH, 1:1000
(mouse, sc-32233)
40 kDa
Colorimetric blot
Bioluminescence blot (exposure: 130s)
dKO-19M-8M
dKO-19-8M
dKO-19M-8
dKO-19-8
Rv1-Luc
-
+
-
+
-
+
-
+
-
+
SNX631 (500nM)
CDK19
CDK8
pSTAT1-S727
total STAT1
GAPDH

## Slide 3
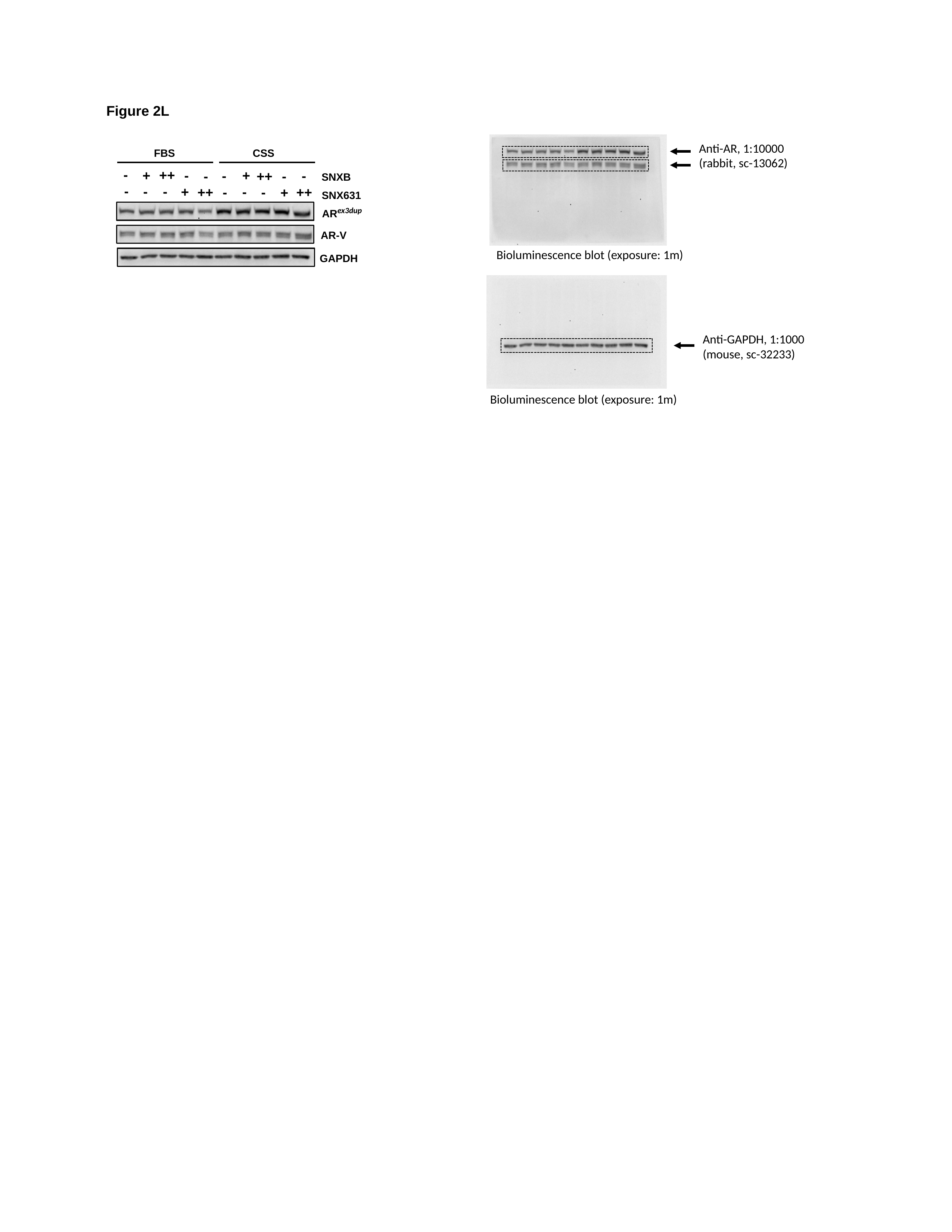

Figure 2L
Anti-AR, 1:10000
(rabbit, sc-13062)
FBS
CSS
-
+
++
-
+
++
-
-
-
-
SNXB
-
-
-
+
-
-
-
++
++
+
SNX631
ARex3dup
AR-V
GAPDH
Bioluminescence blot (exposure: 1m)
Anti-GAPDH, 1:1000
(mouse, sc-32233)
Bioluminescence blot (exposure: 1m)
